# Integrated microbiota–host–metabolome approaches reveal adaptive ruminal changes to prolonged high-grain feeding and phytogenic supplementation in cattle

**DOI:** 10.1093/femsec/fiae006

**Published:** 2024-01-27

**Authors:** Sara Ricci, Cátia Pacífico, Susanne Kreuzer-Redmer, Ezequias Castillo-Lopez, Raul Rivera-Chacon, Arife Sener-Aydemir, Giacomo Rossi, Livio Galosi, Lucia Biagini, Heidi E Schwartz-Zimmermann, Franz Berthiller, Nicole Reisinger, Renee M Petri, Qendrim Zebeli

**Affiliations:** Christian Doppler Laboratory for Innovative Gut Health Concepts of Livestock, Institute of Animal Nutrition and Functional Plant Compounds, Department for Farm Animals and Veterinary Public Health, University of Veterinary Medicine Vienna, Veterinärplatz 1, 1210 Vienna, Austria; Christian Doppler Laboratory for Innovative Gut Health Concepts of Livestock, Institute of Animal Nutrition and Functional Plant Compounds, Department for Farm Animals and Veterinary Public Health, University of Veterinary Medicine Vienna, Veterinärplatz 1, 1210 Vienna, Austria; Christian Doppler Laboratory for Innovative Gut Health Concepts of Livestock, Institute of Animal Nutrition and Functional Plant Compounds, Department for Farm Animals and Veterinary Public Health, University of Veterinary Medicine Vienna, Veterinärplatz 1, 1210 Vienna, Austria; Christian Doppler Laboratory for Innovative Gut Health Concepts of Livestock, Institute of Animal Nutrition and Functional Plant Compounds, Department for Farm Animals and Veterinary Public Health, University of Veterinary Medicine Vienna, Veterinärplatz 1, 1210 Vienna, Austria; Christian Doppler Laboratory for Innovative Gut Health Concepts of Livestock, Institute of Animal Nutrition and Functional Plant Compounds, Department for Farm Animals and Veterinary Public Health, University of Veterinary Medicine Vienna, Veterinärplatz 1, 1210 Vienna, Austria; Christian Doppler Laboratory for Innovative Gut Health Concepts of Livestock, Institute of Animal Nutrition and Functional Plant Compounds, Department for Farm Animals and Veterinary Public Health, University of Veterinary Medicine Vienna, Veterinärplatz 1, 1210 Vienna, Austria; School of Biosciences and Veterinary Medicine, University of Camerino, Via Circonvallazione, 93/95, 62024 Matelica, MC, Italy; School of Biosciences and Veterinary Medicine, University of Camerino, Via Circonvallazione, 93/95, 62024 Matelica, MC, Italy; School of Biosciences and Veterinary Medicine, University of Camerino, Via Circonvallazione, 93/95, 62024 Matelica, MC, Italy; Christian Doppler Laboratory for Innovative Gut Health Concepts of Livestock, Institute of Bioanalytics and Agro-Metabolomics, Department of Agrobiotechnology (IFA-Tulln), University of Natural Resources and Life Sciences, Konrad-Lorenz-Straße 20, 3430 Tulln an der Donau, Austria; Christian Doppler Laboratory for Innovative Gut Health Concepts of Livestock, Institute of Bioanalytics and Agro-Metabolomics, Department of Agrobiotechnology (IFA-Tulln), University of Natural Resources and Life Sciences, Konrad-Lorenz-Straße 20, 3430 Tulln an der Donau, Austria; dsm-firmenich, Animal Health and Nutrition R&D Center, Technopark 1, 3430 Tulln an der Donau, Austria; Agriculture and Agri-Food Canada, Sherbrooke Research and Development Centre, 2000 College Street, Sherbrooke, Quebec J1M 0C8, Canada; Christian Doppler Laboratory for Innovative Gut Health Concepts of Livestock, Institute of Animal Nutrition and Functional Plant Compounds, Department for Farm Animals and Veterinary Public Health, University of Veterinary Medicine Vienna, Veterinärplatz 1, 1210 Vienna, Austria

**Keywords:** bovine, cross-talk, gut health, inflammation, mucosal barrier function, phytogenic feed additives

## Abstract

Diets rich in readily fermentable carbohydrates primarily impact microbial composition and activity, but can also impair the ruminal epithelium barrier function. By combining microbiota, metabolome, and gene expression analysis, we evaluated the impact of feeding a 65% concentrate diet for 4 weeks, with or without a phytogenic feed additive (PFA), on the rumen ecosystem of cattle. The breaking point for rumen health seemed to be the second week of high grain (HG) diet, with a dysbiosis characterized by reduced alpha diversity. While we did not find changes in histological evaluations, genes related with epithelial proliferation (*IGF-1, IGF-1R, EGFR*, and *TBP*) and *ZO-1* were affected by the HG feeding. Integrative analyses allowed us to define the main drivers of difference for the rumen ecosystem in response to a HG diet, identified as *ZO-1, MyD88*, and genus *Prevotella* 1. PFA supplementation reduced the concentration of potentially harmful compounds in the rumen (e.g. dopamine and 5-aminovaleric acid) and increased the tolerance of the epithelium toward the microbiota by altering the expression of *TLR-2, IL-6*, and *IL-10*. The particle-associated rumen liquid microbiota showed a quicker adaptation potential to prolonged HG feeding compared to the other microenvironments investigated, especially by the end of the experiment.

## Teaser

The complex multifunctional ruminal environment in cattle reveals adaptive mechanisms in response to a sustained dietary challenge, which are aided by a phytogenic supplementation.

## Introduction

Ruminants largely depend on the activity of the rumen microbiota to digest and utilize complex dietary polysaccharides for the production of energy, protein, and the maintenance of a healthy rumen ecosystem, owing to the symbiotic host–microbiome relationship (Deusch et al. 2017). The rumen itself is a complex environment, and many studies have highlighted the importance of various niches, hosting a variety of different microorganisms [solid-associated (SAM), liquid-associated (LAM), and epithelial-adherent (EAM) microbiota], differing in preferred substrate for degradation and metabolism. This differential diversity combined with niche interaction is what allows for an optimal utilization of ingested nutrients (De Mulder et al. 2016, Ricci et al. 2022). Despite a significant body of work, our understanding of the metabolic role of these ruminal microbial niches and the extent by which they are affected by diet remains limited, also due to the fact that they are often investigated separately.

A comprehensive characterization of the diet × microbiota interactions and the way they affect the host is of particular interest for feeding regimes that are based on the inclusion of large amounts of grains, commonly fed to meet the high energy requirements of lactating cows. This type of feeding triggers a series of reactions of the ruminal microbiota, that can switch its activity and composition to process the new substrates, ending up with an increased production of fermentation end products, such as volatile fatty acids (VFAs) (Ametaj et al. 2010, Ricci et al. 2022). When the ruminal homeostasis is compromised due to the accumulation of VFAs and the consequent decrease of pH, the animals risk to experience subacute ruminal acidosis (SARA) (Zebeli et al. 2012). In order to adapt to fluctuations in the pH of the rumen milieu, the epithelium increases its absorptive surface area through the combined expression of genes related to growth and nutrient uptake (Steele et al. 2012a, 2015, Dieho et al. 2016). However, these relatively rapid and profound modifications can alter the integrity of the epithelial barrier, compromising the structure of tight junctions and desmosomes (Steele et al. 2011, Liu et al. 2013, McCann et al. 2016). At the same time, the above mentioned dietary challenges may induce changes in microbiota composition and function, leading to production of other microbe-derived compounds with proinflammatory properties such as biogenic amines, lipopolysaccharides (LPS) or lipoteichoic acid, which can alter epithelial inflammation pathways resulting in both local and systemic inflammation (Zhang et al. 2016, Zhao et al. 2018). These alterations of the microbial composition and activity create a perturbation of the ruminal homeostasis, defined as dysbiosis (Sommer et al. 2017).

Given such premises, a healthy rumen depends on the composition of the microbiota and on the metabolites produced by their activity, as well as on the structural and functional integrity of the epithelium. These dynamics have been extensively studied, but usually focusing on isolated aspects, and mainly dealing with only one niche of the rumen microbiota (Ametaj et al. 2010, Fernando et al. 2010, Petri et al. 2020). To overcome the limitations of previous studies, and to characterize the response of the rumen as a complex multifunctional organ, in this study we characterized the microbiota composition of three ruminal niches, the ruminal metabolome, and the rumen epithelial reaction to a prolonged dietary challenge. By collecting samples consecutively over a period of 5 weeks, we aimed to define the precise moment for the onset of dysbiosis, as well as to evaluate the adaptive capacity of the rumen alone or in association with a natural remedy.

Among the strategies to counteract the deleterious effects of high grain (HG) feeding, the use of phytogenic feed additives (PFA) has been demonstrated to modulate ruminal microbial composition and its activity, both *in vitro* and *in vivo* (Calsamiglia et al. 2007, Bodas et al. 2012). Interestingly, PFA in short-term HG feeding have also been reported to alter inflammatory biomarkers, toxin release, and epithelial gene expression, providing positive and encouraging results, such as reduced LPS and biogenic amines concentration, with consequent mitigation of the inflammatory response (Drong et al. 2017, Humer et al. 2018, Petri et al. 2020). Therefore, although the mechanisms of action of such phytogenic compounds are still not fully understood, it remains to be determined if the use of PFA during prolonged HG feeding could provide beneficial effects for the ruminal environment.

The aim of this study was to investigate the effects of prolonged HG feeding challenge on various niches (SAM, LAM, and EAM) of the ruminal ecosystem, by evaluating the structure and functionality of the epithelium, the composition of the microbiota and the production of metabolites. We also evaluated the effects of a PFA supplementation, consisting of a blend of menthol, thymol, and eugenol, and we hypothesized that the feed additive would support the plasticity and adaptability of the ruminal ecosystem, helping to preserve a healthy ruminal environment. The integration of metabolome and microbiota with a panel of genes selected to provide a comprehensive picture of the epithelial reaction allowed us to characterize the ruminal response to a dietary challenge and a PFA supplementation over a period of 5 weeks, providing novel and useful insights on the critical points of the adaptive processes of this ecosystem.

## Materials and methods

### Experiment design and animal housing

The trial was conducted at the research farm of the University of Veterinary Medicine, Vienna, between June and September 2019. The experimental procedure was approved by the Institutional Ethics and Animal Welfare Committee of the University of Veterinary Medicine Vienna and the Austrian national authority according to the European and Austrian laws for animal experiments (protocol number: BMBWF- 68.205/0003-V/3b/2019). A total of nine Holstein nonlactating cannulated cows (Bar Diamond, Parma, ID, USA) were divided into two groups of four and five animals balanced for body weight (mean body weight: 992 ± 73 kg, mean age: 10.0 ± 0.8 years), in a cross-over design with two experimental runs consisting of 6 weeks each. The animals were fed an only forage diet (baseline), consisting of 75% grass silage, 15% corn silage, and 10% grass hay in dry matter basis for 1 week. Then, the cows were transitioned to a HG diet with 65% concentrate via stepwise daily increments of 10% concentrate over 1 week; the HG diet was fed for the following 4 weeks. Details about the diet composition are given in our companion paper (Rivera-Chacon et al. 2022). In addition, one group of animals received a blended PFA at 400 mg kg^−1^ (dry matter basis) (Digestarom^®^, a mixture of essential oils and extracts including menthol, thymol and eugenol; BIOMIN Holding GmbH, which is part of DSM), and the second group served as control; the groups were inverted between runs, resulting in nine genuine replicates per treatment. The feed additive was dosed through the ruminal cannula during the weeks of forage feeding and adaptation to the HG diet, while during the HG feeding the phytogenic blend was included in the concentrate. In the adaptation week the dosage of additive administered through the cannula was adjusted according to the percentage of concentrate included in the diet to reach the targeted daily intake. Feed was mixed and provided once daily (Trioliet Triomatic T15, the Netherlands), and was available *ad libitum*, together with water and mineral blocks. Every cow had access to a single feed bunk and daily feed intake was recorded automatically (Insentec B.V., the Netherlands). Data for feed intake and ruminal pH are reported in our companion paper (Rivera-Chacon et al. 2022). For the whole duration of the experiment, the animals were housed in a free-stall barn with deep litter cubicles (2.6 × 1.25 m, straw litter).

### Sample collection

Samples were collected at five time points, once at baseline and once per every week of HG. Rumen content and rumen papillae were collected 4 h after the morning feeding. To collect samples of SAM and LAM for microbiota analyses, a handful of digesta was sampled from the ruminal mat and the ventral sac of the rumen (Castillo-Lopez et al. 2014). The liquid (LAM) was filtered through four layers of sterile gauze and collected in a beaker. The solid digesta (SAM) was sampled with sterile tweezers. Rumen fluid for metabolomics was collected from the ventral sac of the rumen using a sterile 20 ml syringe. All samples of rumen content were snap frozen and stored at −80°C. For rumen papillae, the rumen was partially emptied and the biopsies were collected following a method previously described (Wetzels et al. 2016, Pacífico et al. 2022). The tissue samples for microbiota (EAM) and gene expression analyses were snap frozen and then stored at −80°C. Samples for histology were fixed in 4% formalin solution (Liquid Production GmbH, Germany) until further processing. Right after the sampling, the rumen was refilled with its content. Each part of the equipment was washed and thoroughly disinfected after every use.

### DNA extraction, sequencing, and sequences analysis

A total of 90 samples were collected for EAM and SAM over the 5 weeks of experiment, while 89 samples were processed for LAM (one sample was lost due to technical issues). DNA was extracted using DNeasy PowerSoil Kit (Qiagen, Germany) with some modifications. Samples were preprocessed performing mechanical and enzymatic lysis, as described by Ricci et al. (2022). The concentration of DNA extracted from each sample was measured with Qubit Fluorometer 4.0 (Life Technologies Corporation, USA) using the Qubit DNA HS Assay Kit (Invitrogen, Thermo Fisher Scientific, Austria). Target 16S rRNA gene amplicon sequencing was performed in an external laboratory (Microsynth, Switzerland), which also completed demultiplexing, trimming of adaptors, and reads merging. Briefly, the V3–V4 hypervariable regions of the bacterial 16S rRNA gene were amplified with primers 341F-ill (5′-CCTACGGGNGGCWGCAG-3′) and 802R-ill (5′-GACTACHVGGGTATCTAATCC-3′), with an expected product of ∼460 bp (Klindworth et al. 2013). Barcodes and Illumina adaptors were added with 16S Nextera two-step PCR for library preparation. Finally, samples were distributed in equimolar pools that were sequenced using a 250-bp paired-end reads protocol for Illumina MiSeq sequencing platform. Merged reads were inspected for quality using FASTQC (Andrews and Babraham Bioinformatics 2010) and were further analyzed with software QIIME 2 (v. 2020.2) (Bolyen et al. 2019). Sequences were filtered for quality (PHRED score 20) and subsequently denoised with deblur (Amir et al. 2017). Reads were trimmed at 400 nucleotides for SAM and LAM samples, and at 385 nucleotides for EAM samples. Denoising caused the loss of two samples for LAM (*n* = 87) and of one sample for EAM. Furthermore, the latter matrix had three samples below 1000 reads, which were discarded (*n* = 86). All SAM samples passed the quality filtering and denoising (*n* = 90). The output tables were filtered to exclude mitochondrial contamination before taxonomy was assigned with a Naive Bayes classifier trained for the specific 16S rRNA gene target regions against the SILVA 132 99% OTU reference database. To calculate alpha diversity, datasets were rarefied to the lowest read count that would allow to keep the maximum number of samples with a Good’s coverage index above 0.90 (9002 reads for SAM, five samples discarded, *n* = 85; 8324 reads for LAM, four samples discarded, *n* = 83; 6526 reads for EAM, six samples discarded, *n* = 80) (Table S1, Supporting Information). Diversity was calculated with “diversity core-metrics-phylogenetic” function in QIIME 2. Beta diversity was calculated in R, using phyloseq package (1.42.0) (McMurdie and Holmes 2013) for weighted and unweighted UniFrac and vegan package (2.6.4) (Oksanen et al. 2020) for Aitchison distance (based on CLR transformation) (Aitchison et al. 2000).

### RNA extraction, reverse transcription, and qPCR

RNA was extracted using RNeasy Mini Qiacube Kit (Qiagen) with some minor modifications. About 25 mg of papillae were mixed with 350 µl RLT buffer in 2 ml safe lock tubes containing 0.6 g of ceramic beads. After homogenization in a Fastprep-24 instrument (MP Biomedicals, USA), samples were centrifuged at 10 000 × *g* for 1 min. The lysate was transferred to a 2-ml tube and centrifuged again at 14 680 × *g* for 3 min. The following steps were performed as described by the manufacturers, but with centrifugation times increased from 15 to 30 s. After the final addition of 500 µl of buffer RPE, the column was centrifuged at full speed for 1 min to dry the membrane. The filter was placed into a new 1.5 ml tube and was left to dry for 1 min. Finally, 30 µl of RNase-free water were added to the filter and incubated for 1 min. RNA was eluted by centrifugation at 10 000 *g* for 1 min and stored at −20°C. Genomic DNA was removed using DNAse I (Ambion® TURBO DNA free), then RNA integrity was assessed using the Qubit RNA IQ Assay Kit and extracted RNA was quantified with the Qubit RNA HS Assay Kit (Invitrogen, Thermo Fisher Scientific) on the Qubit Fluorometer 4.0 (Life Technologies Corporation). Reverse transcription was performed using the High-Capacity cDNA Reverse Transcription Kit (Applied Biosystems™, Thermo Fisher Scientific, USA), with the addition of 500 µl of RNAse inhibitor (20 000 U, Biozym, Austria) in a thermocycler (Nexus, Eppendorf, Germany) with the following conditions: 25°C for 10 min, 37°C for 2 h, 85°C for 5 min, and finally 4°C. Per each sample, 10 µl of template were mixed with 10 µl of 2x mastermix (2 µl of 10x buffer, 0.8 µl of 25x dNTP mix (100 mM), 2 µl of 10x RT random primers, 0.5 µl RNAse inhibitor, 1 µl MultiScribe RT, and 3.7 µl H_2_O, for a total volume of 10 µl), to which were added 80 µl of RNAse free water, to reach a concentration of 10 ng µl^−1^. Gene expression analyses were performed using CFX96 Touch Real-Time PCR Detection System (BioRad, USA), with 10 µl reaction mix (2 µl cDNA as template, 0.8 µl of 100 nM primers, reverse and forward respectively, and Biozym Blue S'Green qPCR master mix). Thermocycler conditions were set for 3 min at 95°C for initial denaturation, followed by 40 cycles of 95°C for 5 s and 60°C for 30 s. Finally, samples were brought to 95°C for 1 min and to 55°C for 5 s; melt curve analysis was set to increment of 0.5°C cycle^−1^. Per each gene, samples were run in two technical replicates and the mean Ct value was used for further calculations. Negative control and reverse transcription control (minus RT) were included in each assay. To normalize for mRNA content, hypoxanthine phosphoribosyltransferase 1 (*HPRT1*) and tyrosine 3-monooxygenase/tryptophan 5-monooxygenase activation protein zeta (*YWHAZ*) were used as housekeeping genes. Primers for the tested genes are reported in Table 1. Primer design was performed for *IL-6, TLR-4, MyD88, EGFR, IGF-1, HPRT1*, and *YWHAZ* with Primer3Plus (version: 3.2.6) (Untergasser et al. 2012), targeting cDNA regions spanning between two exons (when possible) based on published cow sequences [Ensembl, Genome assembly: ARS-UCD1.2 (GCA_002263795.2)]. Gradient qPCRs were run per each new primer pair for estimation of optimal annealing temperature and validation of primer specificity by melting curve analysis.

**Table 1.** Primers used for gene expression analysis.

Gene symbol	Accession number	Forward primer	Reverse primer	Amplicon size (bp)	Annealing temp (°C)	Efficiency (%)	*R* ^2^	Exon spanning	Reference
***IL-6***	ENSBTAG00000014921	CACCCCAGGCAGACTACTTC	GCATCCGTCCTTTTCCTCCA	184	64	93.3	0.996	Exon2/3	This study
***TLR-2***	NM_174197.2	CTGGCCCTTCCTTCAAACCT	GGGGAAGGCACTGGGTTAAA	215	60	95.3	0.985	Exon 2	Pederzolli et al. (2018)
***TLR-4***	XM_005210586.3	TGGGACCCTTGCGTACAG	ACGGCCACCAGCTTCTG	159	60	101.3	0.992	Exon 1/2	This study
***TNF-α***	NM_173966.3	CAAGTAACAAGCCGGTAGCC	AGATGAGGTAAAGCCCGTCA	153	57.5	100.2	0.982	Exon 3/4	Liu et al. (2013)
***IL-10***	ENSBTAG00000006685	GGACCAACTGCACAGCTTAC	CACGTGCTCCTTGATGTCAG	154	57.5	103.8	0.994	Exon2/3	Petri et al. (2020)
***IFN-γ***	NM_174086.1	GCAGCTCTGAGAAACTGGAGGA	ATGGCTTTGCGCTGGATCT	79	60	99	0.993	Exon3/4	Petri et al. (2019)
***MyD88***	ENSBTAG00000000563	GACGACGTGCTGATGGAACT	CCGGATCATCTCGTGGACAA	111	60	96.4	0.998	Exon1/3	This study
***CD14***	NM_174008.1	ATCCACAGTCCAGCCGACAA	CAGCAGCAGCAGCAGGTAGG	97	60	102.5	0.986	Exon 1/2	Petri et al. (2019)
***CLDN-4***	NM_001014391.2	GTGTTTGGCGTGCYGTTGTY	GGCCTTGGAGCTCTCATCAT	72	60	91.5	0.98	Exon 1	Petri et al. (2019)
***DSG1***	NM_174045.1	AGACAGAGAGCAATATGGCCAGT	TTCACACTCTGCTGACATACCATCT	88	60	95	0.996	Exon 6/7	Steele et al. (2015)
***ZO1 (TJP1)***	AJ313183.1	CGACCAGATCCTCAGGGTAA	AATCACCCACATCGGATTCT	164	60	100	0.99	Exon 3/5	Liu et al. (2013)
***EGFR***	ENSBTAG00000011628	CATCCGAGGAAATGTGCTTT	GTTGCAGAGGACAGGGTTGT	157	60	100.6	0.998	Exon 2/3	This study
***IGF-1R***	XM_002696504	GATCCCGTGTTCTTCTACGTTC	AAGCCTCCCACTATCAACAGAA	101	60	93.4	0.993	Exon 12/13	Steele et al. (2012b)
***IGF-1***	ENSBTAG00000011082	GTTGGTGGATGCTCTCCAGT	CTCCAGCCTCCTCAGATCAC	148	58	100.9	0.998	Exon 3/4	This study
***TBP***	NM_001075742.1	CAGAGAGCTCCGGGATCGT	CACCATCTTCCCAGAACTGAATAT	194	58	86.6	0.988	Exon 2/4	Rekawiecki et al. (2012)
***HPRT1***	NW_005397637.1	TTGTATACCCAATCATTATGCTGAG	ACCCATCTCCTTCATCACATCT	109	58	99.7	0.987	Exon 2/3	This study
***YWHAZ***	NM_174814.2	TGAAAGGAGACTACTACCGCTACTTG	GCTGTGACTGGTCCACAATCC	121	58	103.3	0.99	Exon 3/4	This study

### Metabolomics analyses

Metabolite profiling was performed as described in Ricci et al. (2022). For determination of carboxylic acids, sugar phosphates and sugars, 20 µl aliquots of rumen fluid were shaken with 980 µl of acetonitrile/water (80:20, v/v) at 4°C for 10 min, centrifuged at 14 350 × *g* for 10 min and the supernatants were diluted 10-fold with acetonitrile/water (20:80, v/v). Analysis was performed by anion exchange chromatography on a Dionex Integrion HPIC system coupled to a Q Exactive Orbitrap mass spectrometer (both Thermo Scientific). Compounds were quantified based on external calibration curves established between 3 and 9000 ng ml^−1^. Apparent recoveries (determined by comparing peak areas of ^13^C-labeled internal standards of acetic acid, propionic acid, and butyric acid added prior to work-up with peak areas in pure solvent solutions containing the same concentrations) were close to 100%.

Biogenic amines were determined by high-performance liquid chromatography coupled to tandem mass spectrometry (LC-MS/MS) after derivatization with phenyl isothiocyanate. Sample preparation was carried out in 96-well plates and LC-MS/MS analysis was performed on an Agilent 1290 series UHPLC system (Agilent Technologies, Waldbronn, Germany) coupled to a SCIEX 6500+ QTrap mass spectrometer equipped with a Turbo V ion source (SCIEX, Foster City, CA, USA) as outlined in Ricci et al. (2022). Biogenic amines were quantified on the basis of external calibration curves (0.6–1000 ng ml^−1^ in measurement solution) prepared on the same plate. ^13^C-putrescin was added to every sample and standard solution prior to work-up and was used as internal standard for recovery determination. An in-house prepared sample was worked-up and measured three times on each sample preparation day and served as inter- and intraday quality control sample. Metabolome data for integrative analyses were normalized by row sums and Pareto scaling. Missing values and zeros were replaced by the half of the minimum detection limit per each compound.

### Histology and immunohistochemistry

Papillae biopsies for histology were fixed in neutral buffered formalin (4% v/v) and then transferred to 70% ethanol and cleared in xylene before embedding in paraffin wax. Histological sections, 3-μm thick, were stained with DeadEnd™ Colorimetric TUNEL System (Promega Italia Srl, Italy) for the evaluation of cellular apoptosis. For each sample, three papillae were evaluated and count of the immunolabeled cells was performed on three microscopic fields (Leica DM2500 microscope, Germany). The same samples were immunostained with monoclonal mouse antihuman Cytokeratin antibody, clone AE1/AE3 (diluted 1:200, Agilent, USA), to evaluate the thickness of the keratin layer. This evaluation was performed on three papillae per each sample, on three microscopic fields (the apex of each selected papilla and in both lateral sides). The same three papillae were considered for the measurement of the stratum corneum thickness.

### Statistical analyses

Alpha diversity, metabolite profiles, and histology data were analyzed in SAS (v. 9.4). Normality was checked with PROC UNIVARIATE and the PROC REG procedure was used to calculate a linear regression as well as Cook’s distance (Cook’s D). Values were considered outliers with a Cook’s D above 0.08. A linear mixed model was run with the PROC MIXED procedure, with cow, run, treatment, diet within week, and the interaction between diet and treatment within week as fixed effects. Cow within run and group were random effects. Measurements taken on the same cow at different time points were considered as repeated measure, and *post hoc* Tukey correction for *P*-values was applied. Nonrarefied feature tables were used to compute differential abundance analysis with Microbiome Multivariable Associations with Linear Models (MaAsLin2) package (1.7.3) in R (Mallick et al. 2021). Differential abundance was calculated using Centered Log-Ratio (CLR) normalization and LM method, while False Discovery Rate was calculated with Benjamini–Hochberg method (Benjamini and Hochberg 1995). The model was run with diet, treatment, and week as fixed effects and individual animal and experimental run as random effects. To assess the differential abundance between consecutive weeks in HG, the analysis was repeated on subsets of data considering only the 4 weeks in HG for the control and the PFA groups. The model was run with week as categorical fixed effect, individual animal and experimental run as random effects, and changing the reference level in order to assess the changes between consecutive weeks. Nonparametric MANOVA (PERMANOVA) was used to analyze beta diversity (Anderson 2001), through adonis function of vegan package (Oksanen et al. 2020). Differences were tested for fixed effects of diet, treatment, week, and their interaction. Functional prediction for the microbiota data was performed through Phylogenetic Investigation of Communities by Reconstruction of Unobserved States 2 (PICRUSt2), run using the QIIME2 plugin (v. 2019.10), with the default options (average NSTI was 0.26 for SAM samples, 0.28 for LAM samples, and 0.24 for EAM samples) (Bolyen et al. 2019, Douglas et al. 2020).

Principal components analysis (PCA) of metabolites normalized counts was performed to identify the major responsible for distribution variation between the weeks through package stats (4.2.2) (Millard 2013).

For gene expression data, one sample from Run 2 was excluded because of technical issues. Rosner’s test was applied on the Ct values to detect possible outliers (rosnerTest function of EnvStats package, 2.7.0) (Millard 2013), and a total of three observations were removed from the dataset (for *EGFR, IFN-γ*, and *TLR-2*). Gene relative expression was calculated applying the ΔΔCt method (Pfaffl 2001), using two reference genes (*HPRT1* and *YWHAZ*) to calculate the ΔCt. The external calibrator was the mean ΔCt value of each group (treatment or control) on the first week (baseline) per each run, to normalize data considering the crossover design (Petri et al. 2019). Lastly, the relative expression was calculated as value = 2^− ΔΔCt^. The obtained values per each run were then merged into a single dataset on which statistical analyses were performed. A further statistical test was performed on the relative expression values to identify possible outliers, using the PROC REG procedure of SAS to calculate Cook’s D. Values with a Cook’s D above 0.10 were removed from the dataset for downstream analyses (one observation removed from each gene, with the exception of *IFN-γ, TLR-4, IGF-1, IL-6*, and *IL-10* for which two observations were removed, and *ZO-1* for which four values were removed). The linear mixed model was run with PROC MIXED using the same fixed and random effects described above and with *post hoc* Tukey correction for *P*-values. Significance was considered for *P* ≤ .05 and tendencies were discussed for .05 < *P* ≤ .10.

Data integration between microbiota, metabolome and gene expression was performed using unsupervised and supervised approaches. For the first approach, normalized counts of the identified metabolites were used to compute canonical correspondence analysis (CCA) between metabolites and microbiome data for LAM and SAM and EAM using vegan package. Significance was tested with function anova.cca(). The supervised approach was implemented with the function block.splsda() of package MixOmics (Rohart et al. 2017), which performs a multiblock sPLS-DA (Sparse Partial Least Squares Discriminant Analysis). Before running the analysis, the microbial datasets were filtered to retain features with a relative abundance above 0.01% across the whole dataset and normalized with CLR transformation. The normalized metabolome and gene expression were analyzed in combination with LAM, SAM, and EAM separately and with two different response variables (treatment and week), resulting in six different models (three for each explanatory variable). The optimization of the number of components was obtained through perf() function with 10-fold cross-validation and 50 repetitions while the number of features to retain was obtained with the tune.block.splsda() function with 3-fold cross-validation and 50 repetitions. The results of the analysis were evaluated to establish the correlations between datasets by comparing the coefficients for each of the components of each model. The loadings per each component were also evaluated to identify the most discriminant variables. The ASVs are discussed if they resulted among the most influential loadings in at least two of the model components. Relevance networks were obtained with function network() with a cutoff of 0.7 and implemented for visualization using packages igraph and ggraph (Csárdi and Nepusz 2006, Pedersen 2022). Other graphs were produced with package ggplot2 (Wickham 2016).

## Results

### Microbial diversity

SAM samples generated 3 240 905 reads, grouped in 18 155 amplicon sequence variants (ASVs). In LAM samples, 4 077 333 reads were grouped in 18 623 ASVs, while in the EAM samples 2 589 625 reads were grouped in 12 182 ASVs.

**Figure 1.**
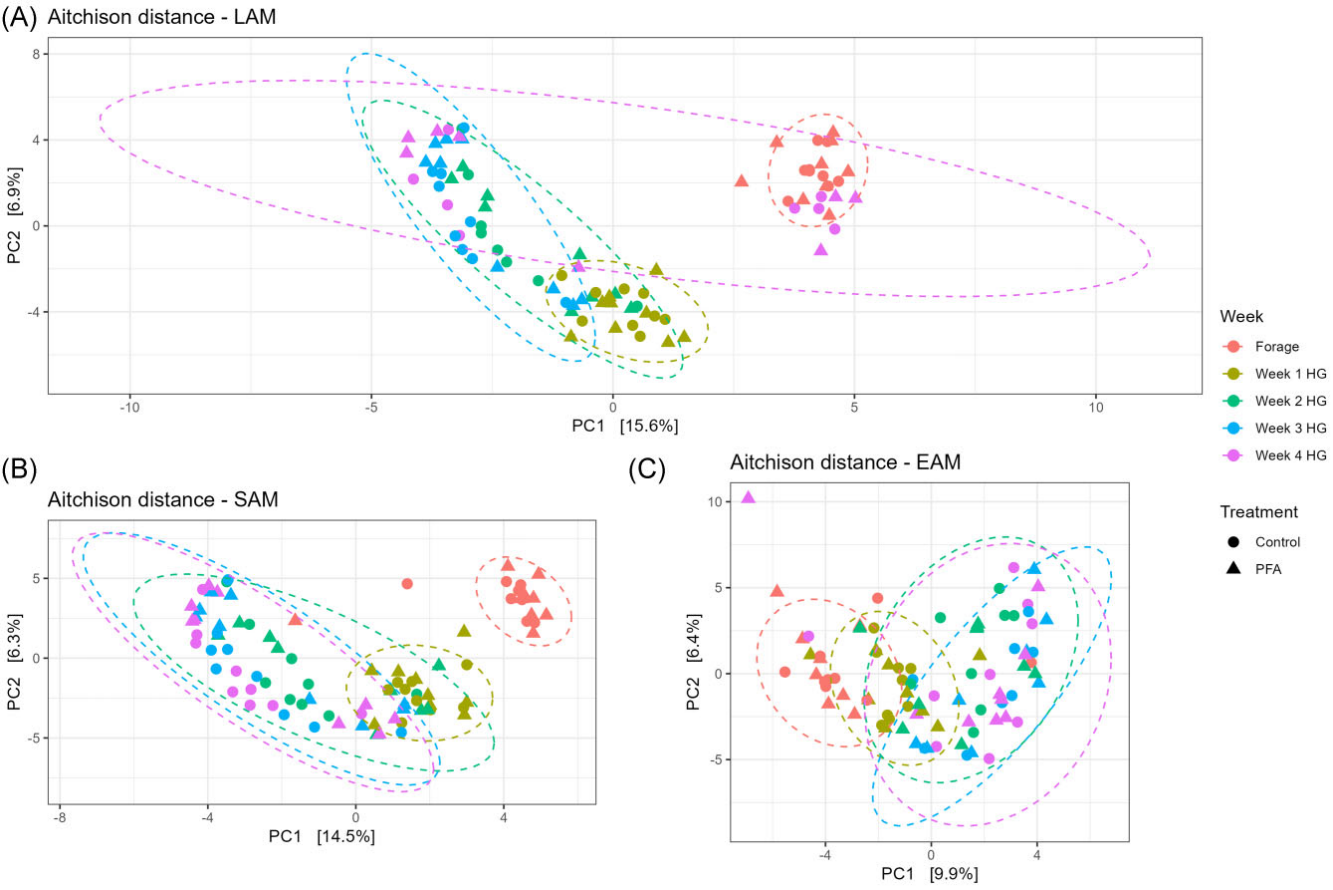
Principal component analysis based on the Aitchison distance measured for three ruminal microenvironments (LAM = liquid associated microbiota; SAM = solid associated microbiota; and EAM = epithelial adherent microbiota) in cows fed a forage-based diet (Forage) and a HG diet for 4 weeks. Results are presented for the control and the treatment (PFA) groups. The distance matrix was affected by the diet and the weeks in all three ruminal niches (*P* < .01 for LAM, SAM, and EAM for both diet and week).

All alpha diversity indices were affected by the diet in LAM and SAM samples (Table 2; Table S1, Supporting Information). In LAM samples, all the alpha diversity indices decreased over the first 2 weeks of HG, and significantly increased in the last 2 weeks of experiment. The same pattern was visible for the SAM samples, with the lowest values reached on the second week of HG, and values numerically increasing in the last week. In EAM samples, Pielou’s evenness and Faith’s Phylogenetic Diversity were affected by the diet (*P* = .05 and *P* = .03, respectively). Shannon index tended to be higher in EAM in the PFA group (*P* = .10). Both weighted and unweighted UniFrac distances were affected by the diet and the experimental week in all the ruminal microenvironments (*P* < .01 for both in LAM and SAM and unweighted UniFrac in EAM; *P* < .01 and *P* = .03 for diet and week, respectively, for weighted UniFrac in EAM). The Aitchison distance matrix results were in agreement with the canonical methods (Fig. 1). The phytogenic treatment did not affect any beta diversity matrices.

**Table 2.** Alpha diversity matrices calculated for solid- (SAM), liquid- (LAM), and epithelial- (EAM) associated microbiota. Mean values and standard error of the mean are presented at the baseline and per each week of HG feeding for control and treatment (PFA) group. (Faith’s PD = Faith’s phylogenetic diversity; Observed ASVs = Observed amplicon sequence variants).

	Forage—baseline		1 HG		2 HG		3 HG		4 HG		*P*-values^1^		
	Control	PFA	Control	PFA	Control	PFA	Control	PFA	Control	PFA	PFA	Diet	I
**SAM**													
**Shannon index**	9.99 ± 0.06^a^	9.84 ± 0.18^a^	8.86 ± 0.15^b^	8.85 ± 0.17^b^	7.37 ± 0.32^a^	7.77 ± 0.34^a^	8.06 ± 0.40	7.66 ± 0.37	8.29 ± 0.23	7.94 ± 0.44	.75	< .01	.71
**Pielou’s evenness index**	0.91 ± <0.01^a^	0.90 ± 0.01^a^	0.85 ± 0.01^b^	0.85 ± 0.01^b^	0.75 ± 0.02^b^	0.77 ± 0.02^b^	0.80 ± 0.02	0.78 ± 0.02	0.81 ± 0.01	0.79 ± 0.03	.95	< .01	.66
**Faith’s PD**	118 ± 3^a^	115 ± 5^a^	84.1 ± 3.5^b^, ^x^	85.3 ± 4.2^b^, ^x^	68.0 ± 5.6^y^	79.0 ± 7.3^y^	79.6 ± 7.4	74.7 ± 7.1	84.7 ± 4.2	78.6 ± 7.9	.74	< .01	.43
**Observed ASVs**	2032 ± 72^a^	1927 ± 128^a^	1360 ± 75^b^	1359 ± 80^b^	925 ± 106^a^	1136 ± 138^a^	1137 ± 154	994 ± 139	1205 ± 100	1106 ± 167	.91	< .01	.34
**LAM**													
**Shannon index**	9.85 ± 0.04^a^	9.91 ± 0.05^a^	8.37 ± 0.23^b^	8.38 ± 0.19^b^	6.29 ± 0.54^a^	6.67 ± 0.41^a^	7.09 ± 0.39^a^	6.93 ± 0.47^a^	8.06 ± 0.75^b^	7.74 ± 0.83^b^	.40	< .01	.74
**Pielou’s evenness index**	0.91 ± <0.01^a^	0.91 ± <0.01^a^	0.83 ± 0.02^b^	0.82 ± 0.01^b^	0.65 ± 0.04^a^	0.69 ± 0.03^a^	0.73 ± 0.03^b^	0.72 ± 0.03^b^	0.78 ± 0.05^a^	0.76 ± 0.06^a^	.38	< .01	.58
**Faith’s PD**	96.6 ± 1.5^a^	96.4 ± 2.1^a^	63.6 ± 3.9^b^, ^x^	65.0 ± 3.1^b^, ^x^	54.1 ± 7.0^y^	55.7 ± 5.5^y^	54.7 ± 4.4^a^	52.7 ± 5.6^a^	76.0 ± 8.0^b^	70.7 ± 10.2^b^	.64	< .01	.95
**Observed ASVs**	1881 ± 43^a^	1909 ± 47^a^	1142 ± 79^b^	1196 ± 76^b^	852 ± 152^a^	862 ± 116^a^	852 ± 98^a^	823 ± 131^a^	1312 ± 220^b^	1238 ± 262^b^	.46	< .01	.98
**EAM**													
**Shannon index**	7.78 ± 0.19	7.82 ± 0.11	8.03 ± 0.14	8.06 ± 0.16	7.65 ± 0.23	8.11 ± 0.10	7.97 ± 0.20	8.13 ± 0.10	7.87 ± 0.21	8.19 ± 0.22	.10	.09	.80
**Pielou’s evenness index**	0.80 ± 0.01	0.81 ± 0.01	0.82 ± 0.01	0.82 ± 0.01	0.80 ± 0.02	0.83 ± 0.01	0.81 ± 0.02	0.82 ± 0.01	0.80 ± 0.02	0.83 ± 0.01	.25	.05	.23
**Faith’s PD**	57.4 ± 3.7	56.4 ± 3.6	58.0 ± 1.9	58.3 ± 2.4	53.5 ± 2.9	58.9 ± 1.1	60.8 ± 2.0	62.3 ± 1.8	60.2 ± 2.0	61.7 ± 3.8	.21	.02	.55
**Observed ASVs**	888 ± 87	848 ± 60	890 ± 47	908 ± 53	770 ± 60	890 ± 22	905 ± 55	933 ± 33	910 ± 54	938 ± 102	.30	.31	.41

1 *P*-values for the effect of phytogenic treatment (PFA), diet within week (Diet) and of the interaction between treatment within week and diet (I).

a, b Values with different superscripts indicate a significant difference (*P* ≤ .05) between consecutive weeks.

x, y Values with different superscripts indicate a tendency for difference (.05 < *P* ≤ .10) between consecutive weeks.

### Microbiota composition and differential abundance in response to the dietary challenge

The dietary challenge caused major changes in the microbiota composition in all the three niches at each taxonomic level evaluated (phylum, family, and genus). The shifts in phyla relative abundances are presented in Figure S1 (Supporting Information). In EAM samples, phylum *Euryarchaeota* (Archaea) tended to increase due to the diet (0.7% in forage and 1.3% in HG, *P* < .01) (Figure S1, Supporting Information). The three ruminal microenvironments reacted differently to the HG diet, with 42, 71, and 18 families significantly affected in the SAM, LAM, and EAM samples, respectively. The three most abundant families in rumen digesta (both SAM and LAM) were *Lachnospiraceae, Ruminococcaceae*, and *Prevotellaceae*, and were all increased from forage to HG (*P* < .01). The most frequent families over all the EAM samples were *Lachnospiraceae* (31.0%), *Ruminococcaceae* (11.1%), *Clostridiales* Family XIII (9.5%), and *Campylobacteraceae* (7.5%). Families *Prevotellaceae* (2.5% in forage and 6.4% in HG, *P* < .01) and *Clostridiales* Family XIII (13.5% in forage and 8.5% in HG, *P* = .03) were affected by the diet.

**Figure 2.**
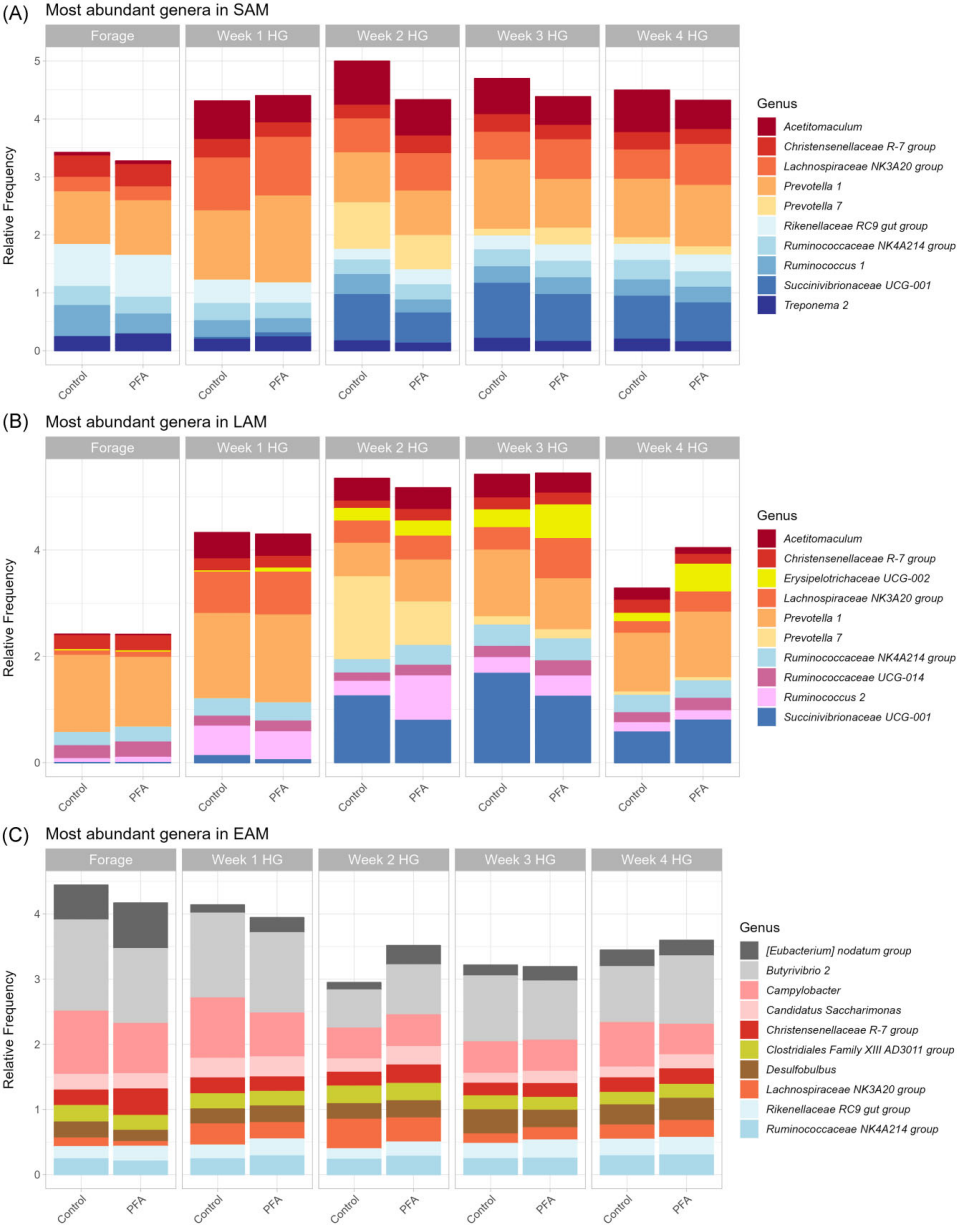
Bar-graph showing the mean relative frequency of the most abundant genera across all SAM (A), LAM (B), and EAM (C) samples. Results are presented for the control and the treatment (PFA) groups for the forage-based diet week (Forage) and for the four HG diet weeks.

**Figure 3.**
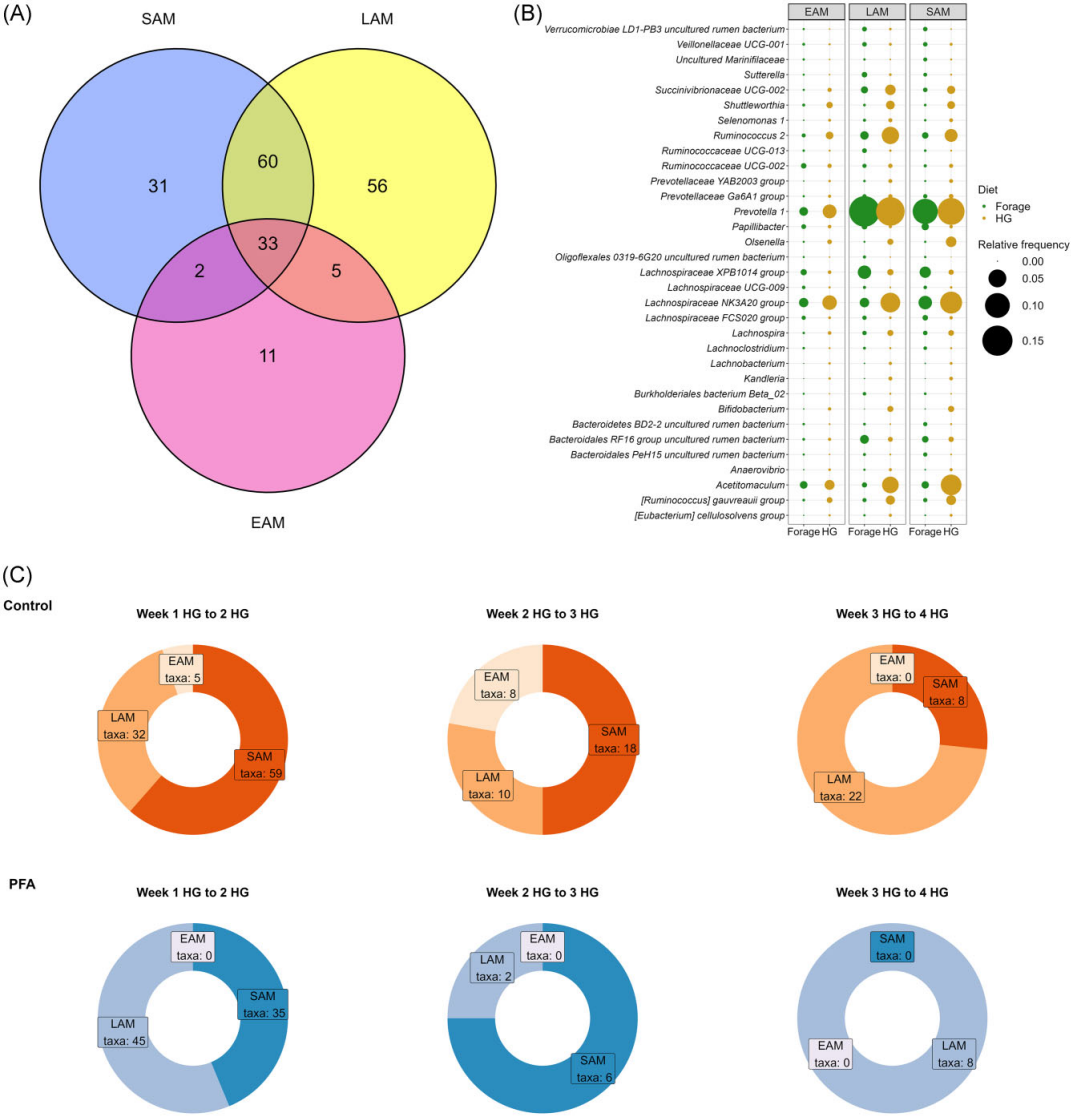
Effect of the HG diet on the ruminal microbiota composition. Venn diagram (A) showing the number of genera differentially abundant due to the HG diet in each ruminal niche analyzed (SAM, LAM, and EAM). Mean relative abundances (B) in forage and in HG feeding of the 33 genera that were affected by the diet in all three ruminal niches. (C) Number of genera differentially abundant during the prolonged HG feeding challenge in each ruminal niche analyzed (SAM, LAM, and EAM). The proportion of significantly different genera between each HG feeding week is represented for the control group and for the treatment group (PFA).

In SAM samples, 461 genera were identified, of which 22 had a relative frequency above 1%. The most abundant genera are presented in Fig. 2(A). In LAM samples, only 21 genera had an overall relative frequency above 1% (Fig. 2B). The most frequent genera over all the EAM samples were classified as *Butyrivibrio* 2 (11.9%), *Campylobacter* (7.5%), [*Eubacterium*] *nodatum* group (3.4%), *Desulfobulbus* (3.1%), and *Ruminococcaceae* NK4A214 group (3.0%) (Fig. 2C). The dietary shift from forage to HG affected 126 and 154 genera in SAM and LAM samples, respectively, while only 51 genera were differentially abundant due to the HG diet in EAM samples (Fig. 3A). Only 33 genera were affected by the HG diet in all three niches (Fig. 3B).

When considering the effect of the duration of the HG dietary challenge 83, 74, and 28 genera were found to be differentially abundant in SAM, LAM, and in EAM samples, respectively. Among the most abundant genera (Fig. 2), *Succinivibrionaceae* UCG-001 was affected by the prolonged HG feeding in all three niches (*P* < .01 in SAM, *P* = .10 in LAM, and *P* = .05 in EAM). Genus *Lachnospiraceae* NK3A20 group was differentially abundant in the experimental weeks in both SAM and LAM samples (*P* = .08 and *P* < .01, respectively). Genera *Ruminococcus* 2 and *Acetitomaculum* were significantly affected by the weeks in LAM samples (*P* < .01), while *Candidatus saccharimonas* tended to be affected by the weeks only in EAM samples (*P* = .07). Genus *Kandleria* was significantly affected by the weeks in all three matrices, reaching the highest concentrations in the first week of HG and decreasing by the end of experiment (*P* < .01 in SAM, LAM, and EAM). Genus *Ruminococcaceae* UCG-002 tended to decrease at the beginning of the HG challenge in all three niches, but reached the highest relative abundance by the end of experiment in SAM and LAM samples (*P* < .01), and on week 3 HG in EAM samples (*P* = .08). On the contrary, *Lachnobacterium* increased in all three niches in the first week of HG feeding and tended to decrease by the end of the HG challenge in LAM and EAM samples (*P* < .01). In SAM samples, *Lachnobacterium* decreased between week 2 (0.013%) and 3 HG (0.009%), to increase again by week 4 HG (0.016%) (*P* < .01). Genus *Acidaminococcus* showed different trends in each microenvironment analyzed. In SAM, it was not present in the first 2 weeks of experiment, and showed the highest relative abundance in week 2 HG (0.47%, *P* < .01). In LAM, *Acidaminococcus* disappeared from forage feeding to week 1 HG, and then increased again to reach the peak on week 3 HG (0.28%, *P* = .01). In EAM samples, the relative abundance of *Acidaminococcus* tended to increase gradually with the progression of the dietary challenge, from a relative abundance < 0.01% in the first week of HG feeding to reach a relative abundance of 0.19% in week 4 HG (*P* = .06).

When specifically analyzing the differential abundance between each consecutive HG week, the differences between the ruminal microenvironments were even more deepened, with distinct genera affected in each niche (Fig. 3C). Genera *Schwartzia* and *Prevotella* 7 were the only two taxa with a significant differential abundance between weeks 1, 2, and 3 HG in all the three niches, reaching the highest relative frequency on week 3 HG in all the microenvironments analyzed. In LAM, family *Bacillaceae* was significantly reduced due to the duration of the HG diet. LAM samples showed a higher reactivity between week 3 HG and week 4 HG compared to the other two ruminal niches analyzed, with higher proportions of differentially abundant genera in both the control and PFA groups (Fig. 3C). The microbiota attached to the rumen wall showed the most stable composition over the 4 weeks of HG diet, with only a few genera significantly different between each week, and only in the control group (Fig. 3C).

### Effect of the PFA on the ruminal microbiota

The PFA supplementation in SAM samples increased the overall relative frequency of uncultured *Peptococcaceae* (0.06% in PFA and 0.03% in control, *P* = .04), *Desulfuromonas* (0.013% in PFA and 0.008% in control, *P* = .02) and *Desulfovibrio* (0.18% in PFA and 0.11% in control, *P* = .06). The treatment tended to decrease the overall relative frequency of genus *Coprococcus* 2 (0.13% in PFA and 0.23% in control, *P* = .08), *Lachnospiraceae* NC2004 group (0.05% in PFA and 0.06% in control, *P* = .09), *Lachnospiraceae* UCG-001 (0.06% in PFA and 0.12% in control, *P* = .06), and *Ruminococcus* 1 (3.1% in PFA and 3.9% in control, *P* = .06). The PFA also decreased the abundance of [*Eubacterium*] *xylanophilum* group (0.12% in PFA and 0.21% in control, *P* < .01) and *Pseudobutyrivibrio* (0.25% in PFA and 0.35% in control, *P* < .01). The PFA supplementation affected 13 predicted pathways in SAM samples (Figure S2, Supporting Information). Overall, more taxa were affected by the prolonged dietary challenge in the control group compared to the PFA, indicating a more stable composition over the 4 weeks of HG feeding (Fig. 3C). No effect was recorded in the PFA group in the transition between week 3 and 4 HG.

In LAM samples, *Lachnospiraceae* NK3A20 group tended to increase in the PFA group (5.7%) compared to control (4.5%) (*P* = .07). Similarly, *Ruminiclostridium* 9 (0.3% in PFA and 0.1% in control, *P* < .01), *C. saccharimonas* (1.3% in PFA and 0.10% in control, *P* = .05), and uncultured *Peptococcaceae* (0.03% in PFA and 0.01% in control, *P* = .06) were more frequent in the PFA group. [*Eubacterium*] *xylanophilum* group (0.14% in PFA and 0.17% in control, *P* = .10), and *Lachnospiraceae* NC2004 group (0.03% in PFA and 0.04% in control, *P* = .04) were more frequent in the control group. The PFA supplementation also affected 24 predicted pathways in LAM samples (Figure S2, Supporting Information). Like for the SAM, in LAM samples the PFA group showed a more stable composition compared to the control group, with less genera differentially abundant between the four consecutive weeks in HG (Fig. 3C).

The PFA supplementation did not affect the microbiota composition nor the predicted activity in the EAM samples.

### Metabolite profiling

**Figure 4.**
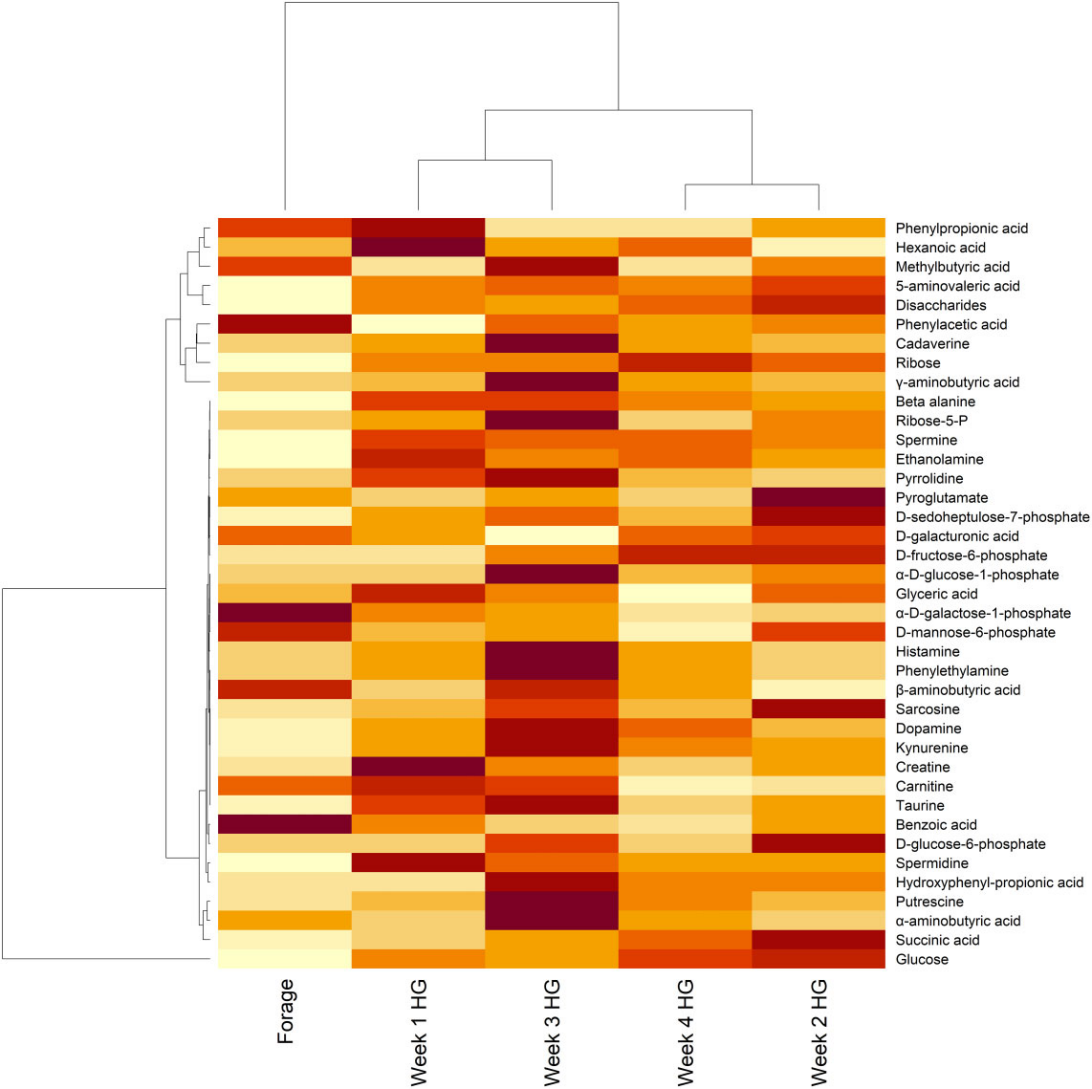
Heatmap showing the change in concentration of metabolites measured in the rumen fluid across the five experimental weeks. Rows were scaled to have mean zero and standard deviation one, to normalize the different concentrations of each metabolite. Values range from −1 (lowest concentration measured) to 1 (highest concentration measured). The hierarchical clustering shows a separation between the baseline (Forage) and HG feeding weeks (1–4 HG).

**Figure 5.**
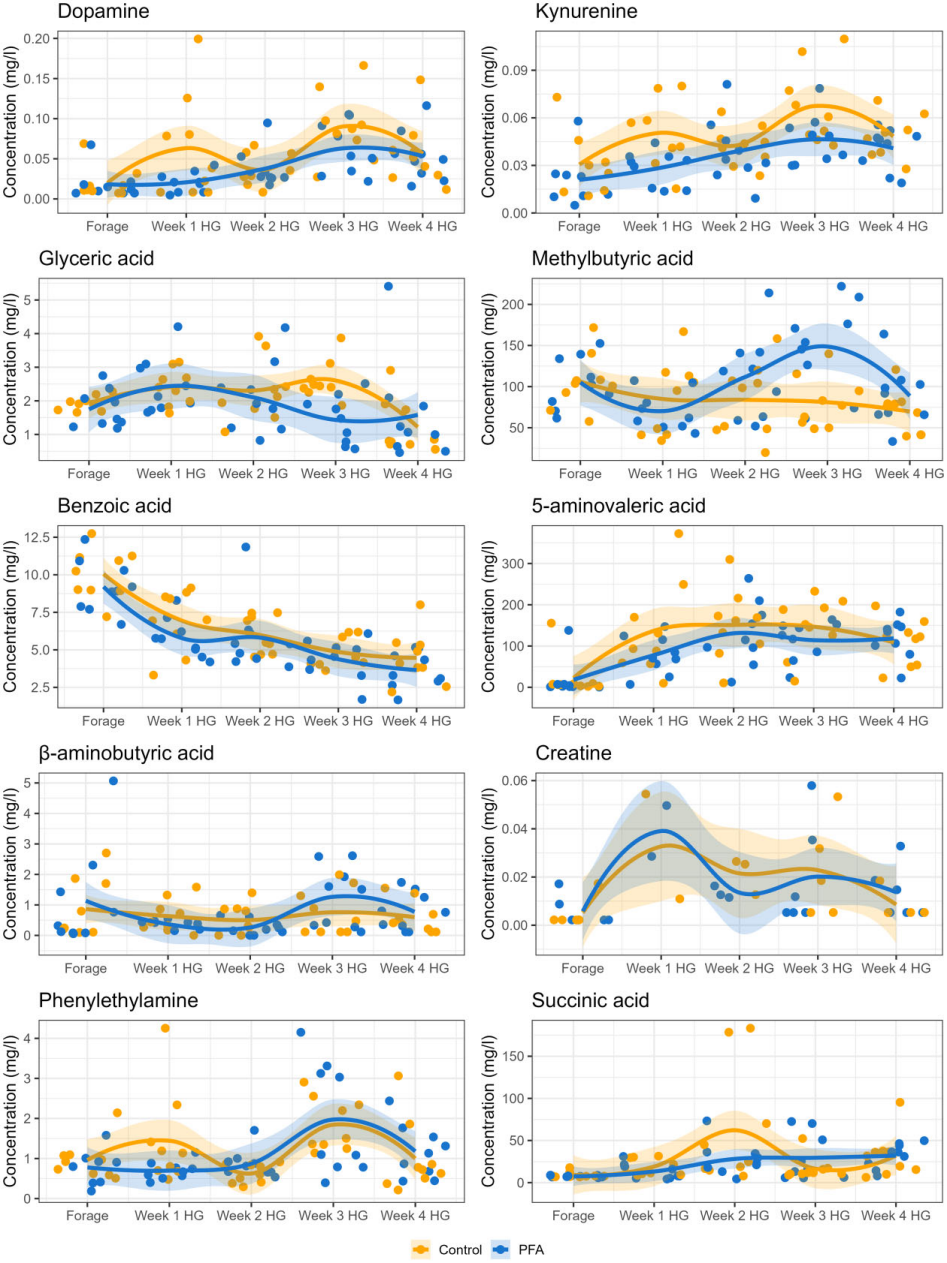
Variation in concentration over the experimental weeks of the metabolites affected by the phytogenic treatment (PFA). Concentrations were measured in the rumen fluid of cows fed a forage-based diet (Forage) and a HG diet for 4 weeks. Results are presented per each metabolite for the control and the PFA groups per each experimental week.

**Figure 6.**
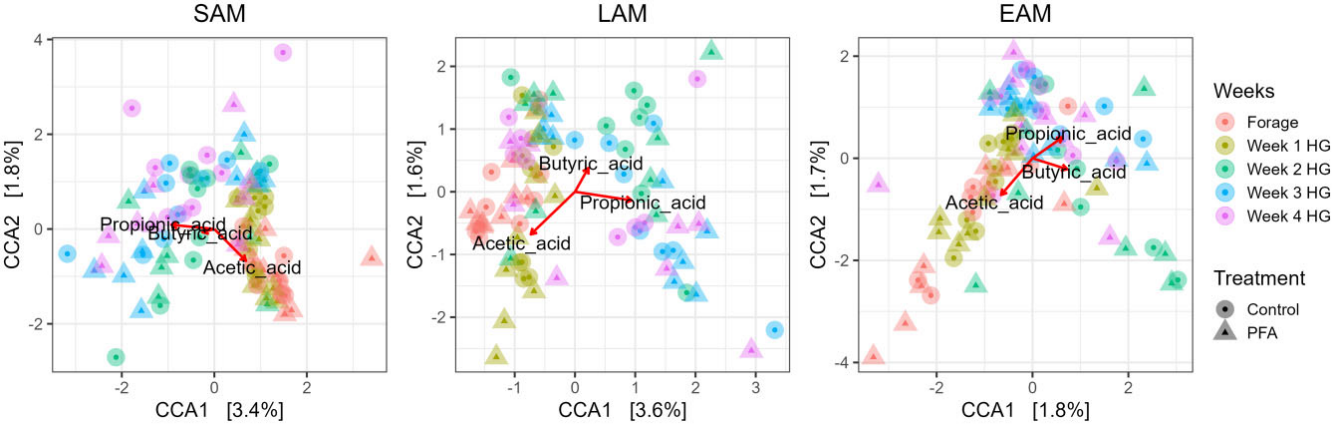
CCA plot. CCA was performed between microbiome data for SAM, LAM, EAM, and the three metabolites identified as main responsible for the variation in ruminal metabolites concentration. Results are presented for the control and the treatment (PFA) groups for the forage-based diet week (Forage) and for the four HG diet weeks.

All the metabolites measured in the rumen fluid, apart from carnitine, ethylbutyric acid, pyroglutamate, α-d-glucose-1-phosphate, and hydroxyphenyl-propionic acid, were affected either by the diet or by the interaction between the treatment and the diet (Table 3). The variation of metabolite concentration over the weeks is shown in Fig. 4. The PFA maintained or tended to maintain a stable lower level of dopamine (*P* = .08), kynurenine (*P* = .01), glyceric acid (*P* = .02), and benzoic acid (*P* = .03) compared to the control. There was an interaction between the treatment and the diet for six of the analyzed metabolites: 5-aminovaleric acid (5-AVA) (*P* = .02), β-aminobutyric acid (BABA) (*P* = .04), creatine (*P* = .09), phenylethylamine (*P* = .07), methylbutyric acid (*P* = .01), and succinic acid (*P* = .09) showed a great variation of concentration in the different weeks. In particular, the PFA supplementation seemed to stabilize the concentrations of 5-AVA, kynurenine and succinic acid over the experimental weeks (Fig. 5). PCA confirmed the three major VFAs (acetic, propionic, and butyric acid) as major responsible for the metabolite distribution variation between the weeks (Figure S3, Supporting Information). CCA performed between normalized counts of these three VFAs and the microbiome data revealed a significant impact for all three niches (*P* < .01). Significance was tested also per each VFA, revealing a significant influence of each acid, despite the low variance explained (*P* < .01, *P* = .01, and *P* = .03 for propionic, acetic, and butyric acid, respectively in SAM; *P* < .01, *P* < .01, and *P* = .03 for propionic, acetic and butyric acid, respectively in LAM; *P* = .03, *P* = .03, and *P* = .01 for propionic, acetic, and butyric acid, respectively in EAM) (Fig. 6).

**Table 3.** Metabolites measured in rumen fluid. Mean values and standard error of the mean are presented at the baseline and per each week of HG feeding for control and treatment (PFA) group. Concentration is given in µg ml^−1^.

	Forage—baseline		1 HG		2 HG		3 HG		4 HG		*P*-values^1^		
	Control	PFA	Control	PFA	Control	PFA	Control	PFA	Control	PFA	PFA	Diet	I
**γ-aminobutyric acid**	32.0 ± 30.6	32.2 ± 31.3	103 ± 48	19.1 ± 17.2	39.4 ± 15.7	80.2 ± 30.6	250 ± 58	125 ± 55	77.5 ± 36.9	74.5 ± 28.5	.29	<.01	.30
**5-aminovaleric acid**	22.5 ± 16.6	18.2 ± 15.0	137 ± 38	77.8 ± 15.3	151 ± 28	131 ± 26	147 ± 24^c^	114 ± 16^d^	109 ± 19	119 ± 16	.34	<.01	.02
**α-aminobutyric acid**	18.2 ± 15.9^a^	13.2 ± 12.3^a^	14.4 ± 8.6^b^	4.20 ± 2.14^b^	9.15 ± 4.73	13.0 ± 5.6	64.4 ± 22.6	4.86 ± 0.72	18.9 ± 8.3	15.3 ± 6.5	.47	.01	.26
**ß-aminobutyric acid**	0.86 ± 0.33	1.14 ± 0.55	0.63 ± 0.18	0.37 ± 0.04	0.50 ± 0.17^a^	0.26 ± 0.08^a^	0.76 ± 0.25^b^	1.26 ± 0.33^b^	0.60 ± 0.18	0.77 ± 0.20	.23	<.01	.04
**Acetic acid**	5749 ± 373^a^	5061 ± 552^a^	7043 ± 724^b^	7904 ± 739^b^	6673 ± 560	7659 ± 787	6791 ± 270	6912 ± 483	7088 ± 715	6361 ± 481	.86	.03	.83
**α-d-galactose-1-phosphate**	2.32 ± 0.09	2.30 ± 0.11	2.06 ± 0.23^x^	1.80 ± 0.23^x^	1.60 ± 0.39^y^	1.68 ± 0.34^y^	1.44 ± 0.34	2.12 ± 0.63	1.54 ± 0.56	1.67 ± 0.32	.38	<.01	.42
**α-d-glucose-1-phosphate**	1.86 ± 0.10	1.88 ± 0.13	1.82 ± 0.19	1.93 ± 0.18	1.67 ± 0.35	2.42 ± 0.62	2.15 ± 0.62	2.51 ± 0.44	1.78 ± 0.34	2.14 ± 0.84	.94	.25	.63
**Benzoic acid**	10.1 ± 0.6^a^	9.20 ± 0.58^a^	6.95 ± 0.69^b^	5.78 ± 0.43^b^	5.99 ± 0.42	5.81 ± 0.80	4.86 ± 0.33	4.38 ± 0.44	4.47 ± 0.58	3.65 ± 0.40	.03	<.01	.89
**Beta alanine**	1.79 ± 0.64^a^	1.27 ± 0.62^a^	4.19 ± 1.05^b^	2.08 ± 0.52^b^	2.31 ± 0.43	2.54 ± 0.53	3.82 ± 0.80	2.45 ± 0.36	2.40 ± 0.52	3.17 ± 0.81	.55	<.01	.32
**Butyric acid**	949 ± 82^a^	849 ± 75^a^	2088 ± 348^b^, ^x^	2384 ± 195^b^, ^x^	1602 ± 143^y^	1897 ± 127^y^	1581 ± 68	1778 ± 235	1801 ± 114	1705 ± 288	.27	<.01	.44
**Cadaverine**	22.8 ± 10.8^a^	14.8 ± 7.9^a^	59.7 ± 14.5^b^	26.2 ± 11.1^b^	24.4 ± 6.3^x^	36.7 ± 12.0^x^	116 ± 14^y,a^	97.0 ± 26.4^y,a^	34.4 ± 20.1^b^	48.0 ± 15.4^b^	.33	<.01	.32
**Carnitine**	0.02 ± 0.01	0.02 ± <0.01	0.01 ± <0.01	0.02 ± 0.01	0.01 ± <0.01	0.02 ± <0.01	0.02 ± <0.01	0.02 ± <0.01	0.01 ± <0.01	0.01 ± <0.01	.15	.16	.41
**Creatine**	0.01 ± <0.01	0.01 ± <0.01	0.03 ± 0.02	0.04 ± 0.01	0.02 ± <0.01	0.01 ± <0.01	0.02 ± 0.01	0.02 ± 0.01	0.01 ± <0.01	0.01 ± <0.01	.73	.23	.09
**d-fructose-6-phosphate**	1.14 ± 0.23	1.27 ± 0.18	0.85 ± 0.30^x^	0.99 ± 0.35^x^	2.09 ± 0.84^y^	5.94 ± 2.01^y^	1.76 ± 0.47	3.54 ± 1.17	1.33 ± 0.10	7.95 ± 7.73	.89	.05	.75
**d-galacturonic acid**	4.12 ± 0.44	3.32 ± 0.28	3.67 ± 0.71	3.00 ± 0.50	3.85 ± 0.85^a^	3.88 ± 1.12^a^	1.76 ± 0.49^b^	2.28 ± 0.69^b^	4.59 ± 1.93	2.88 ± 1.40	.56	<.01	.52
**d-glucose-6-phosphate**	4.14 ± 0.21	4.28 ± 0.13	4.19 ± 0.55^a^	3.86 ± 0.48^a^	6.16 ± 1.36^b^	13.1 ± 4.0^b^	5.98 ± 0.95^b^	10.19 ± 2.25^b^	3.23 ± 0.67^a^	5.87 ± 3.60^a^	.58	<.01	.19
**d-mannose-6-phosphate**	3.46 ± 0.16^a^	3.40 ± 0.16^a^	2.36 ± 0.19^b^	2.23 ± 0.14^b^	2.46 ± 0.53	4.14 ± 1.13	2.04 ± 0.26^a^	3.14 ± 0.58^a^	0.95 ± 0.21^b^	2.15 ± 1.42^b^	.50	<.01	.31
**d-sedoheptulose 7-phosphate**	1.58 ± 0.26	1.54 ± 0.26	3.45 ± 0.76	3.36 ± 1.58	4.44 ± 2.87	6.32 ± 3.15	4.05 ± 0.50	4.47 ± 2.02	2.89 ± 1.46	3.40 ± 1.29	.97	.09	.50
**Dopamine**	0.02 ± 0.01	0.02 ± 0.01	0.06 ± 0.02	0.02 ± <0.01	0.04 ± 0.01^a^	0.04 ± 0.01^a^	0.09 ± 0.01^b^	0.06 ± 0.01^b^	0.06 ± 0.01	0.05 ± 0.01	.08	<.01	.53
**Ethanolamine**	0.48 ± 0.09^a^	0.38 ± 0.12^a^	3.09 ± 0.70^b^	3.62 ± 0.68^b^	1.99 ± 0.21	1.94 ± 0.33	1.85 ± 0.55	3.00 ± 1.15	2.50 ± 0.65	2.56 ± 0.73	.66	<.01	.11
**Glucose**	41.4 ± 6.2^a^	78.1 ± 11.4^a^	541 ± 131^b^	445 ± 93^b^	801 ± 101	667 ± 131	428 ± 27	441 ± 97	701 ± 137	634 ± 183	.96	<.01	.66
**Glyceric acid**	1.93 ± 0.07	1.75 ± 0.19	2.41 ± 0.18	2.44 ± 0.28	2.30 ± 0.31	2.10 ± 0.35	2.61 ± 0.19^a,c^	1.43 ± 0.24^a,d^	1.22 ± 0.26^b^	1.58 ± 0.51^b^	.02	<.01	.07
**Histamine**	0.60 ± 0.37	0.30 ± 0.19	1.49 ± 0.49	0.86 ± 0.50	0.32 ± 0.08^a^	0.51 ± 0.13^a^	5.81 ± 1.37^b^	3.15 ± 0.95^b^	0.89 ± 0.48^a^	1.23 ± 0.29^a^	.26	<.01	.43
**Hexanoic acid**	88.9 ± 5.8	78.7 ± 11.4	100 ± 14^a^	136 ± 18^a^	62.8 ± 14.3^b^	77.9 ± 16.0^b^	97.0 ± 18.8	77.0 ± 7.8	86.3 ± 14.7	107 ± 26	.75	.03	.46
**Hydroxyphenyl-propionic acid**	5.67 ± 1.30	5.22 ± 1.13	6.51 ± 1.50	4.62 ± 0.45	10.40 ± 3.55	9.35 ± 1.04	15.2 ± 8.3	13.2 ± 5.3	6.27 ± 1.58	14.1 ± 7.0	.33	.21	.88
**Iso-butyric acid**	128 ± 13^a^	120 ± 9^a^	81.8 ± 4.3^b^	84.0 ± 6.4^b^	74.1 ± 8.2^b^	87.4 ± 8.7^b^	99.5 ± 6.2^a^	101 ± 7^a^	92.3 ± 6.0	86.8 ± 5.2	.36	<.01	.45
**Iso-valeric acid**	148 ± 13^a^	132 ± 8^a^	89.9 ± 11.2^b^	83.6 ± 8.5^b^	89.8 ± 10.7	102 ± 13	110 ± 7	107 ± 11	102 ± 9	84.9 ± 4.0	.53	<.01	.60
**Kynurenine**	0.03 ± 0.01	0.02 ± 0.01	0.05 ± 0.01	0.03 ± <0.01	0.04 ± <0.01	0.04 ± 0.01	0.07 ± 0.01	0.05 ± 0.01	0.05 ± <0.01	0.04 ± <0.01	.01	<.01	.46
**Methylbutyric acid**	106 ± 11^a^	105 ± 11^a^	84.9 ± 15.2^b^	70.2 ± 7.8^b^	84.0 ± 14.8	112 ± 17	81.0 ± 10.4^a,c^	149 ± 18^a,d^	69.7 ± 8.7^b^	88.7 ± 12.3^b^	.08	<.01	.01
**Putrescine**	1.98 ± 1.14^a^	1.91 ± 1.24^a^	13.5 ± 3.2^b^	10.8 ± 2.8^b^	12.6 ± 0.8^b^	15.3 ± 4.5^b^	41.6 ± 6.6^a^	36.4 ± 8.5^a^	20.7 ± 4.2	22.9 ± 6.1	.61	<.01	.87
**Phenylethylamine**	0.96 ± 0.16	0.78 ± 0.14	1.44 ± 0.40	0.70 ± 0.07	0.63 ± 0.08^a^	0.87 ± 0.12^a^	1.84 ± 0.22^b^	1.97 ± 0.47^b^	1.00 ± 0.30^a^	1.18 ± 0.22^a^	.53	<.01	.07
**Pyrrolidine**	2.85 ± 0.17^a^	2.46 ± 0.33^a^	5.12 ± 0.40^b^	4.21 ± 0.37^b^	2.66 ± 0.40^a^	2.68 ± 0.25^a^	4.79 ± 0.63^b^	5.58 ± 0.45^b^	3.16 ± 0.39^a^	2.97 ± 0.31^a^	.91	<.01	.18
**Phenylacetic acid**	72.8 ± 7.6^a^	66.5 ± 3.5^a^	33.6 ± 7.3^b^	31.0 ± 4.8^b^	51.2 ± 4.6^a^	57.7 ± 5.3^a^	58.6 ± 4.1	58.2 ± 6.1	51.7 ± 6.2	47.1 ± 3.9	.83	<.01	.83
**Phenylpropionic acid**	106 ± 8	97.7 ± 13.0	103 ± 10	111 ± 8	88.8 ± 8.0	94.3 ± 4.1	86.8 ± 5.4	79.3 ± 7.0	87.8 ± 7.0	76.1 ± 6.2	.90	<.01	.69
**Propionic acid**	916 ± 80^a^	846 ± 125^a^	1610 ± 144^b^	1737 ± 105^b^	2573 ± 301^a^	2195 ± 295^a^	1906 ± 168	2025 ± 273	1703 ± 160	1670 ± 250	.71	<.01	.56
**Pyroglutamate**	3.43 ± 0.53	3.60 ± 0.91	3.51 ± 0.75	2.32 ± 0.61	7.17 ± 2.31	4.18 ± 1.62	3.68 ± 1.08	3.09 ± 0.62	2.33 ± 0.64	3.54 ± 0.77	.89	.73	.58
**Ribose 5-P**	1.58 ± 0.48	1.85 ± 0.54	2.10 ± 0.78	2.37 ± 0.68	2.43 ± 0.75	2.37 ± 0.66	3.73 ± 1.10^a^	3.22 ± 0.93^a^	2.65 ± 1.70^b^	0.96 ± <0.01^b^	.69	.01	.58
**Pentoses**	11.1 ± 2.1^a^	14.1 ± 3.5^a^	64.5 ± 13.2^b^	59.4 ± 10.6^b^	74.9 ± 9.8	71.5 ± 14.3	54.1 ± 8.1	69.3 ± 14.5	88.9 ± 15.4	81.9 ± 17.4	.69	<.01	.18
**Spermidine**	1.23 ± 0.26^a^	1.24 ± 0.28^a^	16.9 ± 2.7^b^	15.6 ± 3.1^b^	8.98 ± 1.36^a^	7.86 ± 1.23^a^	12.3 ± 4.4	12.3 ± 1.8	9.47 ± 2.09	9.09 ± 1.62	.47	<.01	.42
**Spermine**	0.19 ± 0.03^a^	0.19 ± 0.03^a^	3.96 ± 0.93^b^	3.18 ± 0.85^b^	2.84 ± 0.75	2.36 ± 0.68	2.66 ± 0.97	3.57 ± 1.10	3.36 ± 0.91	3.22 ± 0.86	.60	<.01	.86
**Sarcosine**	0.17 ± 0.03	0.16 ± 0.05	0.27 ± 0.06	0.14 ± 0.02	0.33 ± 0.09	0.39 ± 0.06	0.32 ± 0.04	0.30 ± 0.08	0.25 ± 0.06	0.16 ± 0.03	.45	.03	.27
**Succinic acid**	9.11 ± 1.23^x^	7.43 ± 0.34^x^	19.0 ± 3.2^y^	13.2 ± 3.1^y^	62.2 ± 23.3	28.2 ± 6.6	16.6 ± 4.6	29.5 ± 9.2	32.0 ± 8.9	32.1 ± 4.8	.86	<.01	.09
**Disaccharides**	8.04 ± 2.40^a^	13.8 ± 2.4^a^	121 ± 33^b^	86.5 ± 19.7^b^	171 ± 25	149 ± 33	93.5 ± 6.9	105 ± 19	144 ± 28	118 ± 35	.98	<.01	.61
**Taurine**	0.03 ± 0.01	0.03 ± 0.01	0.11 ± 0.03	0.08 ± 0.02	0.07 ± 0.01	0.06 ± 0.01	0.12 ± 0.03	0.11 ± 0.04	0.05 ± 0.01	0.04 ± 0.01	.36	.01	.44
**Valeric acid**	181 ± 10^a^	156 ± 21^a^	258 ± 30^b^	307 ± 24^b^	296 ± 32^b^	286 ± 14^b^	276 ± 50^a^	235 ± 16^a^	239 ± 14	247 ± 28	.84	<.01	.89

1 *P*-values for the effect of phytogenic treatment (PFA), diet within week (Diet) and of the interaction between treatment within week and diet (I).

a, b Values with different superscripts indicate a significant difference (*P* ≤ .05) between consecutive weeks.

x, y Values with different superscripts indicate a tendency for difference (.05 < *P* ≤ .10) between consecutive weeks.

c, d Values with different superscripts indicate a significant difference (*P* ≤ .05) between Control and treatment (PFA) groups within the same week.

### Gene expression and histology

**Figure 7.**
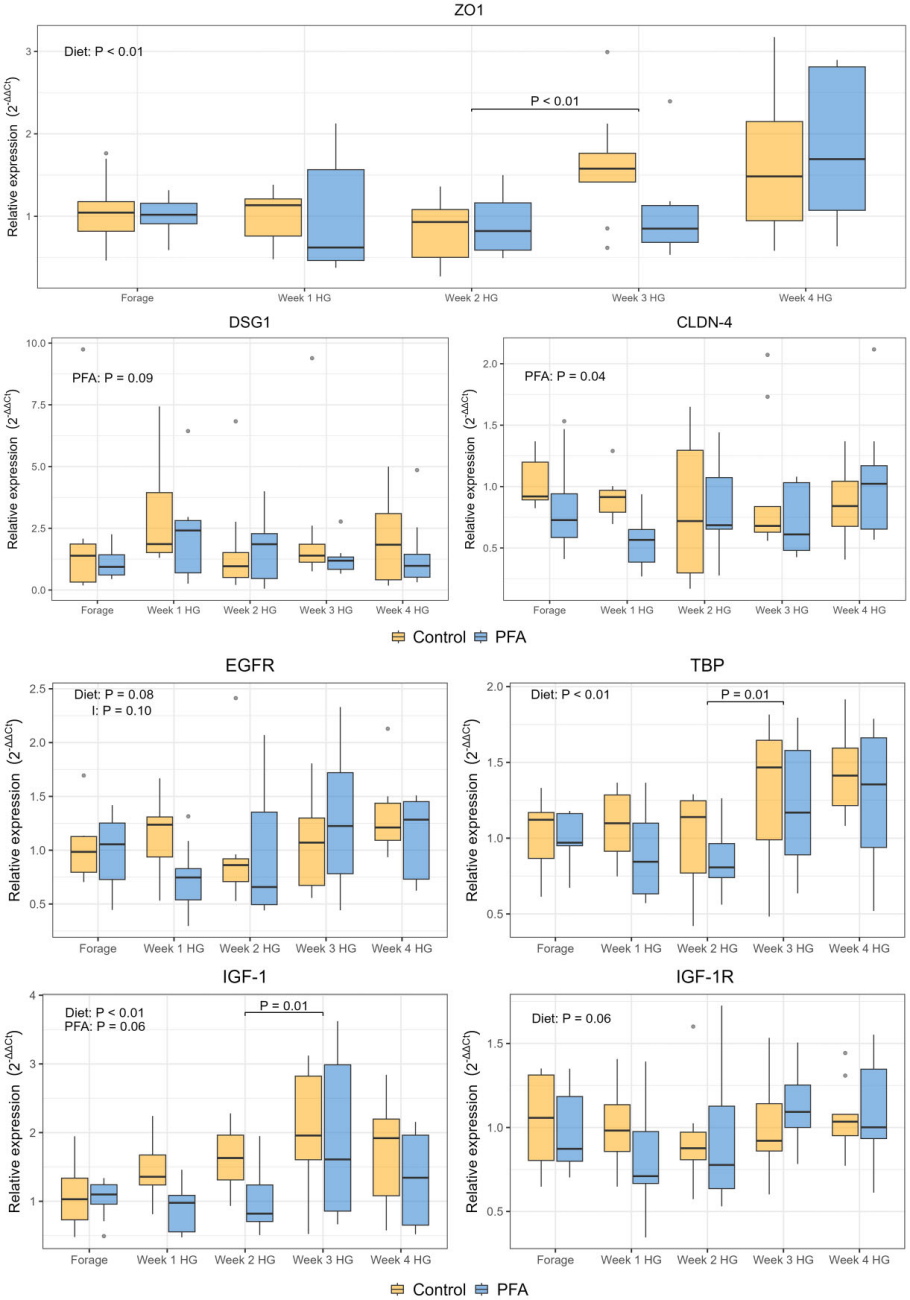
Boxplot showing the relative expression of genes related with barrier function, epithelial growth, and cellular activity in the rumen epithelium of cows fed a forage-based diet (Forage) and a HG diet for 4 weeks. Results are presented for the control and the treatment (PFA) groups per each experimental week. Significant results are presented in each graph for the overall effect of the diet within week (Diet), of the phytogenic treatment (PFA), and of the interaction between treatment within week and diet (I). *P*-values are also presented for the significant pairwise comparisons between consecutive weeks and between control and PFA treatment within each week.

Gene expression analysis showed an effect of the PFA supplementation on the relative expression of two genes related with barrier function [desmoglein 1 (*DSG1*) and claudin-4 (*CLDN-4*)], with higher values for the control group (*DSG1, P* = .09 and *CLDN-4, P* = .04). Zonula occludens-1 (*ZO-1*) was affected by the HG diet (*P* < .01), with a significant increment between week 2 HG and week 3 HG (*P* < .01) (Fig. 7).

All epithelial growth-related genes were affected by the prolonged HG feeding (Fig. 7). Insulin-like growth factor 1 (*IGF-1*) increased between week 2 HG and week 3 HG (*P* = .01) and tended to be higher in the control group (*P* = .06). There was a trend for interaction between diet and PFA treatment for epidermal growth factor receptor (*EGFR, P* = .10), while both *EGFR* and insulin-like growth factor 1 receptor (*IGF-1R*) tended to be affected by the diet (*P* = .08 and *P* = .06, respectively). Tata-box binding protein (*TBP*) was affected by the diet (*P* < .01).

**Figure 8.**
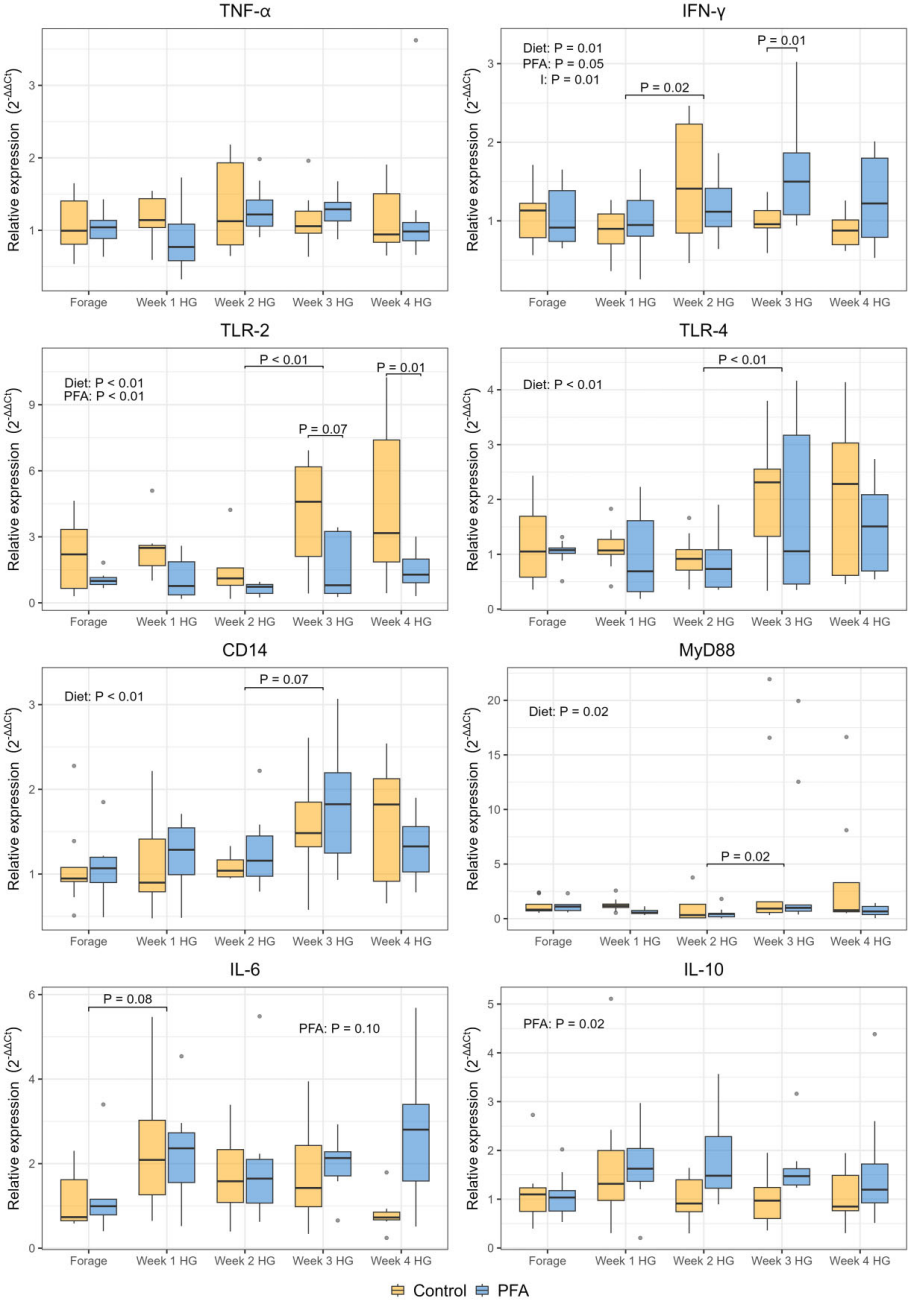
Boxplot showing the relative expression of genes related with inflammation function in the rumen epithelium of cows fed a forage-based diet (Forage) and a HG diet for 4 weeks. Results are presented for the control and the treatment (PFA) groups per each experimental week. Significant results are presented in each graph for the overall effect of the diet within week (Diet), of the phytogenic treatment (PFA), and of the interaction between treatment within week and diet (I). *P*-values are also presented for the significant pairwise comparisons between consecutive weeks and between control and PFA treatment within each week.

While tumor necrosis factor-α (*TNF-α*) was not affected by the weeks in HG, nor by the treatment, interferon-gamma (*IFN-γ*) showed a higher expression in the PFA group (*P* = .05) (Fig. 8). *IFN-γ* was also affected by the diet (*P* = .01) and increased between week 1 HG and 2 HG (*P* = .02). Cluster of differentiation 14 (*CD14*) and toll-like receptor 4 (*TLR-4*) were both affected by the diet (*P* < .01), as well as myeloid differentiation primary response 88 (*MyD88*) (*P* = .02). Toll-like receptor 2 (*TLR-2*) increased over the weeks, especially between week 2 HG and 3 HG (*P* < .01) and was more expressed in the control group (*P* < .01), especially in the last 2 weeks of experiment (Fig. 8). Interleukin-6 (*IL-6*) and interleukin (*IL-10*) showed a higher expression in the PFA group (*P* = .10 and *P* = .02, respectively).

Statistical analyses revealed no effects on the histological parameters evaluated, apart from a tendency for an effect of the individual cow on the thickness of the stratum corneum (*P* = .10) (Table S2, Figures S4 and S5, Supporting Information).

### Data integration analysis

The multiblock sPLS-DA revealed high correlations between microbiota and metabolome, regardless of the chosen explanatory variable (treatment or week) (Tables S3 and S4, Supporting Information). The strongest correlation was observed for LAM for both explanatory variables. This microbial niche also showed the lowest correlation with the gene expression data. In general, the gene expression data showed overall lower correlation coefficients with the other datasets.

**Figure 9.**
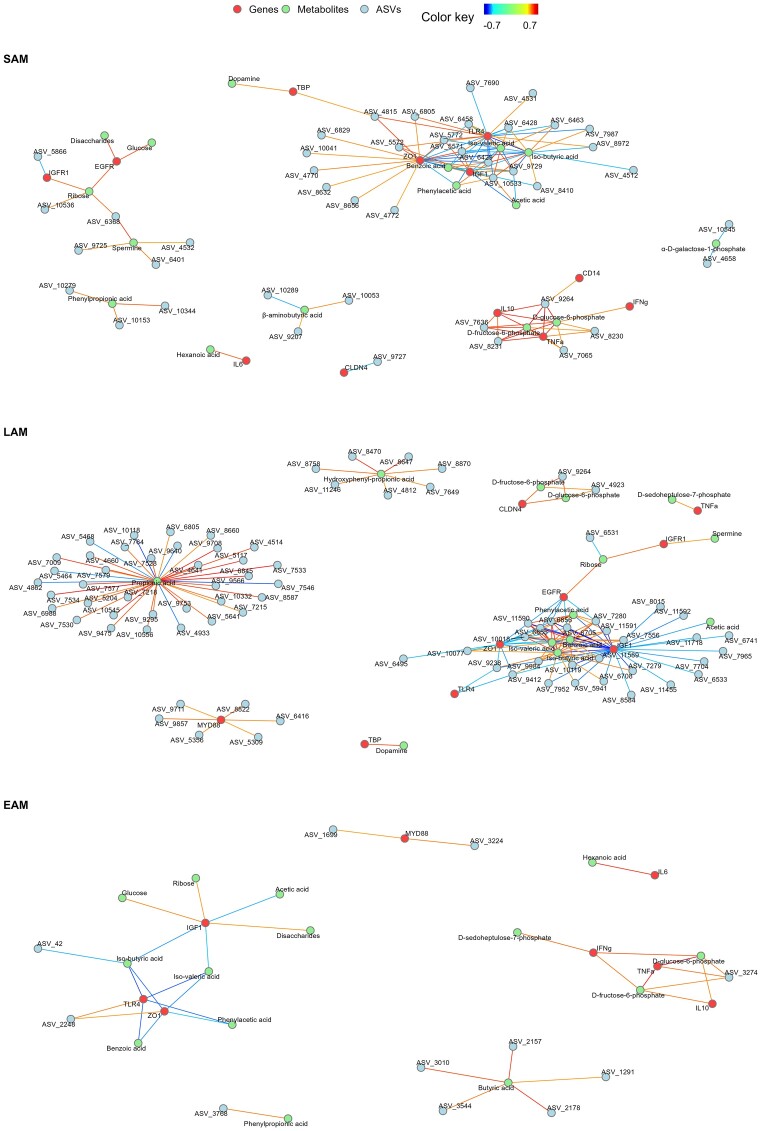
Relevance networks obtained from the sPLS-DA using week as explanatory variable. The networks integrate the gene expression (genes), metabolome (metabolites), and microbiota (ASVs) datasets for SAM, LAM, and EAM, respectively. The associations are shown for a cut off ≥ |0.7|.

**Figure 10.**
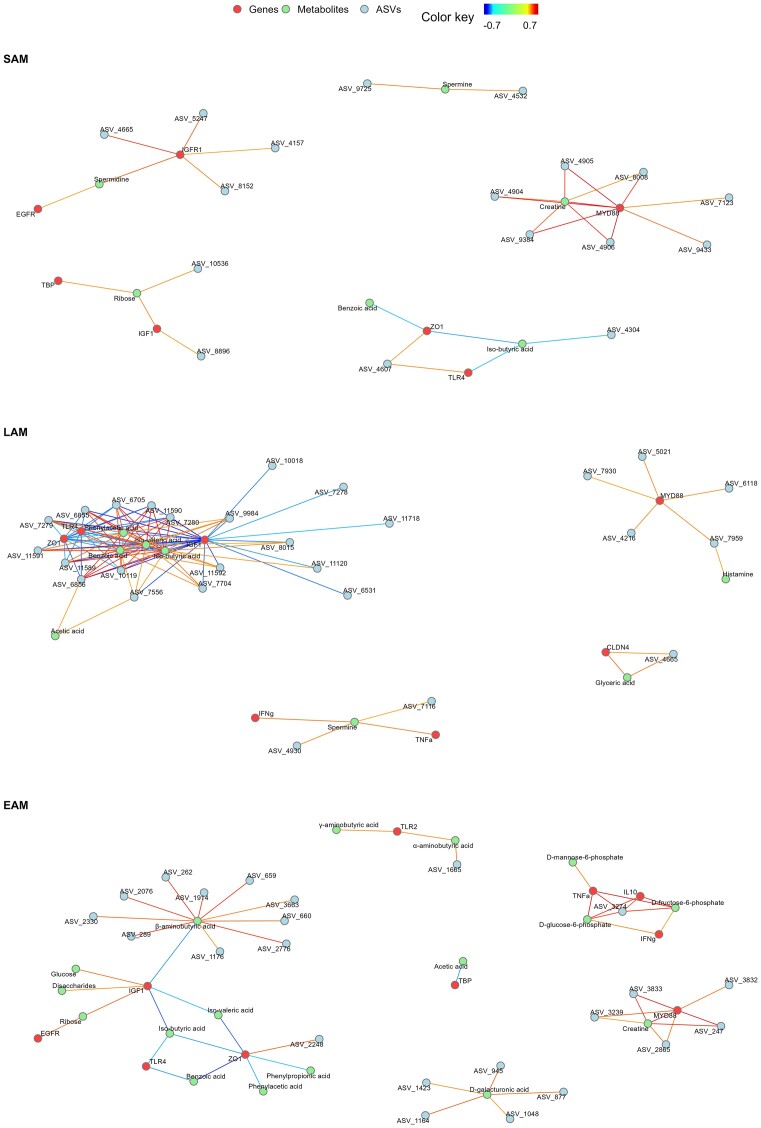
Relevance networks obtained from the sPLS-DA using treatment (PFA) as explanatory variable. The networks integrate the gene expression (genes), metabolome (metabolites), and microbiota (ASVs) datasets for SAM, LAM, and EAM, respectively. The associations are shown for a cut off ≥ |0.7|.

Both the loadings and the relevance networks obtained per each microbial dataset and the two response variables were thoroughly examined to find the key discriminant features. The loadings for each model are presented in Figures S6–S8 (Supporting Information), and the relevance networks are shown in Figures 9 and 10. The classification of the ASVs included in the model is provided in Table S5 (Supporting Information).

### sPLS-DA results for the weeks

In the model considering SAM and weeks as explanatory variable, only eight ASVs were identified in two components. Three were classified as *Prevotella* 1, and other two as *Prevotellaceae* UCG-003 and UCG-004. In the model for LAM and weeks, 15 ASVs were identified in more than one component, of which five were belonging to family *Prevotellaceae*. For EAM, 16 ASVs were found in more than one component, and two were classified as *Prevotella*. ASVs classified as *Rikenellaceae* RC9 gut group and *Christensenellaceae* R-7 group were found to be among the most influential in EAM and in SAM and LAM, respectively.

Genes *CLDN4, DSG1, IGF-1, IL-6, MyD88*, and *TBP* were among the most influential discriminants between weeks for SAM. Similarly, genes *DSG1, IGF-1, IL-6*, and *MyD88* were among the most influential in the model considering LAM. Other influential genes for this model were *ZO-1* and *IGF-1R*. In the model considering EAM, most of the genes were considered discriminant in several components of the model, while genes *TLR-2* and *MyD88* were considered the most influential.

For the metabolome data in the models considering week as explanatory variable, the most influential compounds for SAM were succinic acid, phenylpropionic acid, kynurenine, hexanoic acid, d-glucose-6-phosphate, d-fructose-6-phosphate, and butyric acid. When LAM was included in the model, the metabolites that contributed the most to the differences between groups were propionic acid and histamine. For EAM, the most influential metabolites were identified as hexanoic acid, d-galacturonic acid, and butyric acid.

The relevance networks obtained using week as explanatory variable reinforced the findings obtained by confronting the loadings per each model. Only a few correlations were found for genes related with inflammation although some inflammatory genes (*IFN-γ, IL-10*, and *TNF-α*) were consistently correlated in all the networks. Similarly, *TLR-4, ZO-1*, and *IGF-1* were negatively correlated in all three networks. Both *IGF-1* and *ZO-1* were highly correlated with many other variables. Glucose and disaccharides were positively correlated with *EGFR* and *IGF-1* in the relevance networks obtained for SAM and EAM, respectively. Acetic acid was negatively correlated with *IGF-1* in all the networks. d-glucose-6-phosphate and d-fructose-6-phosphate were present in all the relevance networks and highly correlated with *CLDN4, IFN-γ, IL-10*, and *TNF-α*. Three ASVs classified as *Ruminococcaceae* were positively correlated with d-glucose-6-phosphate and d-fructose-6-phosphate, *IL-10* and *TNF-α* in the network for SAM. Benzoic acid, iso-butyric acid, iso-valeric acid, phenylacetic acid, and acetic acid were also present and highly correlated in all three networks and were consistently associated with epithelial barrier or epithelial growth genes (especially *ZO-1* and *IGF-1*). Propionic acid was identified as major component of the relevance network only in LAM, showing correlations with 36 ASVs. Only ASV_6805 (*Solobacterium*), and ASV_9264 (*Ruminococcaceae* UCG-014) were shared between LAM and SAM networks, the latter showing a strong correlation with d-glucose-6-phosphate and d-fructose-6-phosphate in both datasets.

### sPLS-DA results for the PFA

The sPLS-DA was unable to consistently identify microbial features responsible for the variation among treatment and control groups in both SAM and LAM. For EAM, six ASVs were identified as main drivers of difference (Figure S8, Supporting Information).

In the model considering SAM, *ZO-1* was identified by several components as discriminant feature for the PFA. Other discriminant genes for this niche were *MyD88, IGF-1, IGF-1R*, and *CLDN4*. Similarly, when LAM was included in the model, *ZO-1* was identified among the most important genes for discrimination among groups, together with *IL-10, TLR-4, MyD88, IGF-1, EGFR*, and *CD14*. When EAM was considered in the model, most of the genes were identified as discriminant in more than five components. The highest contributions were found for *MyD88* and *DSG1*.

For the models considering the PFA as explanatory variable and SAM, the highest contribution for the metabolome was given by glyceric acid and creatine, while considering LAM, succinic acid, spermine, and glyceric acid were the most discriminant metabolites. When considering EAM in the model, only few metabolites were found to be main drivers of difference in more than one component. The highest contributions were given by α- and β-aminobutyric acid, hydroxyphenilpropionic acid, and d-galacturonic acid.

Similar patterns were identified between the relevance networks based on week or treatment explanatory variables. Genes related to epithelial growth or barrier integrity showed stronger correlations compared to the inflammation related genes. Gene *MyD88* showed positive correlations with creatine in the networks for EAM and SAM and was correlated with several ASVs in all three networks. For SAM, positive correlations were found with ASV_4906, ASV_4905, ASV_4904 (all classified as *Prevotella* 1), and ASV_9384 (*Ruminococcaceae* UCG-014). Similarly, in LAM network *MyD88* was positively correlated with ASV_5021 (*Prevotella* 1), ASV_7959, and ASV_7930 (both classified as *Ruminococcaceae* UCG-010). In EAM, *MyD88* showed correlations with ASV_3832, ASV_3239, ASV_3833 (*Lachnospiraceae*), and ASV_2865 (*Ruminococcaceae* CAG-352).

*TLR-2* in the network for EAM was positively associated with γ- and α-aminobutyric acid, which was in turn correlated with ASV_1665 (*Rikenellaceae* RC9 gut group). In the same network, β-aminobutyric acid was negatively correlated with *IGF-1* and with 10 ASVs. ASV_4665 (*Prevotella* 1) was found both in SAM and LAM, but strongly correlated with *IGF-1R* in the former and with glyceric acid and *CLDN4* in the latter. In SAM, spermine was positively correlated with ASV_4532 (*Prevotellaceae* UCG-004) and ASV_9725 (*Acetitomaculum*). In LAM, positive correlations for spermine were found with *IFN-γ, TNF-α*, ASV_4930 (*Prevotella* 1), and ASV_7116 (*C. saccharimonas*).

## Discussion

### Impact of HG feeding on the ruminal microbiota composition and activity

We investigated the microbiota composition in three ruminal microenvironments, which have been previously demonstrated to host distinct microbial populations (De Mulder et al. 2016, Wang et al. 2017). As expected, due to HG feeding, alpha diversity was reduced in all the three ruminal niches, with possible implications on impaired animal health (Hook et al. 2011, Plaizier et al. 2017). Our study has also confirmed previous findings, in which the epimural associated microbiota was less responsive to dietary changes (Mann et al. 2018). In fact, in addition to the lower number of taxa affected by the HG diet in this niche, the relevance network for the weeks found few significant correlations between EAM and the other datasets.

In contrast to a previous study, we found a significant impact of the diet on the Archaeal population in the ruminal papillae (Xue et al. 2019). Other studies have reported that methane production is dependent on the percentage and type of concentrates in the diet, suggesting that the increased relative frequency of *Euryarchaeota* could be explained by the composition of our diet (Hook et al. 2010, Moate et al. 2017). However, it also needs to be considered that 16S rRNA gene amplicon sequencing is recognized as a less accurate way to assess the presence of Archaea in the rumen (De Mulder et al. 2016).

According to the unsupervised data integration analysis, the main metabolites responsible for the variation in metabolome composition as well as of the rumen contents microbiota composition were acetic, propionic, and butyric acid. These VFAs are normally the most concentrated in the rumen, and are considered the main microbial fermentation products (Aschenbach et al. 2011). The supervised integrative analyses confirmed propionic and butyric acid as main discriminant of difference between experimental weeks in SAM and especially in LAM.

However, VFAs were not the only metabolites identified as key discriminant by the sPLS-DA. Microbial fermentation, especially in case of dysbiosis, can produce harmful compounds, such as biogenic amines. Of the 20 biogenic amines identified in the present study, kynurenine, phenylethylamine, and histamine were among the most discriminant features for the HG challenge, although we did not find significant correlations between these metabolites and other variables. Some of these compounds, such as histamine and ethanolamine, have been previously reported as increased in cows with SARA, and can contribute to the development of more severe and systemic symptoms (Nocek 1997, Ametaj et al. 2010). Statistical analyses confirmed that all biogenic amines in our study were affected by the diet, except for carnitine and creatine.

Overall, our results suggest a shift towards an imbalanced microbial composition and metabolism due to the HG diet, outlining a picture compatible with ruminal dysbiosis. Since the main drivers for the onset of this condition in the rumen seem to be not only VFAs, but also biogenic amines, such metabolites should be always evaluated when investigating dysbiosis in cattle.

### The effect of dysbiosis on the epithelial expression of inflammation related genes

Toll-like receptors, among others, are receptors responsible of the interaction with bacteria, and can promote tolerance as well as trigger an immune reaction and relative inflammation (Malmuthuge et al. 2012). *TLR-4* recognizes LPS and triggers a reaction that depends on *CD14* and results in the production of pro-inflammatory cytokines. The expression of both genes has been demonstrated to increase in cows with SARA (Stefanska et al. 2018). In accordance with this, *CD14* relative expression increased in parallel with *TLR-4* in response to the HG diet. Interestingly, the relevance networks showed a close connection between *TLR-4* and genes related to epithelial growth and structure (*IGF-1* and *ZO-1*), but not with *CD14*. Such cluster was identified in all the networks and was composed of VFAs and of a different number of ASVs depending on the microbial niche, mostly classified as *Lachnospiraceae* and *Ruminococcaceae*, as well as *Prevotella* 1, and *Rikenellaceae* RC9 gut group. Interestingly, strong correlations were found with family *Bacillaceae* in LAM. Although the other families included in this cluster were increased in all the microbial niches due to the HG diet, *Bacillaceae* decreased in LAM. This family is reputed beneficial for the host and was positively correlated with butyric and iso-butyric acid, benzoic acid, and iso-valeric acid in the cluster. These organic acids have anti-inflammatory properties, which might explain the negative correlations observed with *TLR-4* (Mentschel et al. 2001, Pu et al. 2018, Zhang et al. 2021).

After the activation due to *CD14* and *TLR-4*, the inflammation process can develop following two different pathways. The *MyD88* dependent pathway activates the signaling cascade of NF-kB and consequently of *TNF-α* and other cytokines (Björkbacka et al. 2004). *MyD88* was identified as one of the main drivers of difference between experimental weeks. Its expression was strongly correlated with ASVs belonging to family *Prevotellaceae*, and particularly *Prevotella* 1, indicating the major role played by this Gram-negative family in the development of an inflammatory response in relation to HG. Surprisingly, the increased expression of *MyD88* due to the diet was not accompanied by an increased expression of interleukins in our study. This could be due to the fact that despite the increased expression, especially in some individuals during weeks 2 HG and 3 HG, by the last week of experiment the average cytokines relative expression returned to levels more similar to the forage feeding. The relative expression of genes such as *TLR-4* and *TNF-α*, as well as of other proinflammatory chemokines, was reduced after repeated exposure to LPS (Kent-Dennis et al. 2020). This might indicate the activation of a negative feedback loop and suggests a progressive mechanism of tolerance towards LPS, which could explain the results of our experiment (Lv et al. 2017, Petri et al. 2019). In contrast to our results, a study with a shorter experimental period showed a significant increment of proinflammatory cytokines (Zhang et al. 2016), confirming that the tolerance toward LPS most likely occurs after over 4 weeks of exposure. It needs to be considered that the overall abundance of *Prevotellaceae* increased in the first 2 weeks of HG diet, to decrease again towards the end of experiment: this might also explain the expression of cytokines over the experimental weeks, given the high prevalence of this family in the ruminal environment and the strong association found with *MyD88*. Furthermore, since the levels of LPS were not measured in the present study, it needs to be considered that the concentration might have fluctuated over the weeks also in relation to other Gram-negative bacteria, generating the observed response.

The response to LPS can be mediated also by other proteins, generating a *MyD88* independent pathway (Björkbacka et al. 2004), which results in the production of Type I and Type II interferons (*IFN-γ*) (Mortellaro et al. 2015, Lee and Ashkar 2018). In contrast to the other cytokines, *IFN-γ* relative expression was affected by the diet. Studies *in vitro* and in mouse models suggested that *IFN-γ* might be strongly correlated to dysbiosis and that its expression is more stimulated by Gram-positive bacteria (Hessle et al. 2000, Bae et al. 2020). Therefore, although Gram-negative bacteria and LPS play a widely recognized part in SARA pathogenesis, the role of Gram-positive bacteria in ruminal dysbiosis should be further investigated. This seems to be confirmed also by the strong correlation between ASVs classified as *Ruminococcus* 2 and *Ruminococcaceae* UCG-014 and *IL-10* and *TNF-α*, since both genes were strongly associated to *IFN-γ* in the relevance networks. Notably, d-glucose-6-phosphate and d-fructose-6-phosphate were strongly associated with the same genes, suggesting that the epithelial inflammation associated with the HG diet might be indeed related to glucose metabolism. Glucose is metabolized in glucose-6-phosphate and subsequently in fructose-6-phosphate, and in fact the concentration in the rumen of both metabolites increased in week 2 HG. Thus, it seems to be the metabolization of glucose that triggers the inflammatory reaction of the rumen epithelium, rather than the increment of glucose concentration itself (Peiró et al. 2016). In fact, the activity of glucose-6-phosphate dehydrogenase, which is the rate-liming enzyme for the pentose phosphate pathway, seems to be closely related to the activation of the inflammasome in response to bacterial infections (Yen et al. 2020). Strong positive correlations between these metabolites and ASVs classified as *C. saccharimonas, Ruminococcus* 2, and *Ruminococcaceae* UCG-014 were found for both LAM and SAM. All three genera are mainly amylolytic, suggesting that the production of d-glucose-6-phosphate and d-fructose-6-phosphate likely derived from the increased starch degradation during HG feeding (La Reau and Suen 2018, Baker 2021). The role of glucose-6-phosphate dehydrogenase and glucose-6-phosphate in the onset of the inflammatory response of the ruminal epithelium warrants further research.

### The rumen can adapt to the perturbation caused by long term HG feeding

The adaptive mechanism of papillae enlargement to increase the absorbing surface is finely regulated by several receptors that bind a multitude of proteins. Among these, the insulin-like growth factor axis promotes tissue growth by triggering the production of protein kinases (Kaulfuβ et al. 2009, Steele et al. 2012a). This seems to be validated by our study, since HG feeding caused a higher expression of both *IGF-1* and its receptor (*IGF-1R*). The upregulation of *IGF-1* is associated with an increased uptake of glucose as well as with the proliferation of rumen epithelial cells (Baldwin 1999, Shen et al. 2004, Steele et al. 2012b) and the relevance networks showed correlations of glucose and disaccharides with *EGFR* and *IGF-1*. Therefore, the increased concentration of glucose in the rumen, together with disaccharides, seems to be directly linked to epithelial growth.

The tendency of *IGF-1R* to increase in the last weeks of experiment might indicate a higher cellular metabolic activity towards the end of the trial. This higher activity of the rumen epithelial cells was further suggested by the increased expression of *EGFR* and *TBP* over the weeks. The latter gene is involved in the initiation of the transcription processes, suggesting a higher activity of the cells with the progression of the experiment (Akhtar and Veenstra 2011). Previous studies have demonstrated that some proteins can regulate the expression of *TBP*, although a relationship with the microbiota has never been investigated (Seto et al. 1992, Kerr et al. 1993).

Despite the alterations in epithelial gene expression that we observed due to the diet, and although previous studies documented the adaptations of rumen wall when the animals are fed a HG diet (Steele et al. 2011, 2015), we did not find any modification of the parameters evaluated through histology and immunohistochemistry. It needs to be considered, however, that changes in mRNA expression do not necessarily result in translation into proteins, as there are numerous post-transcriptional mechanisms that could prevent the proteins from being built (Greenbaum et al. 2003, Pacífico et al. 2022). Furthermore, since the samples for histology were collected only in the first and the last weeks of experiment, it is possible that the epithelium had already started recovering, and therefore differences between forage and high-concentrate feeding were not appreciable.

It has been demonstrated that bacterial metabolites can impair the function of the ruminal epithelial barrier (Gao et al. 2022). In our study several biogenic amines of microbial origin, such as cadaverine, dopamine, putrescine, and histamine, showed a peak in concentration during the third week of HG. In the relevance networks, dopamine was positively correlated with *TBP* and spermine with *IGF-1R*, but no correlations with the barrier integrity genes were found. In fact, nor *DSG1* nor *CLDN-4* were affected by the diet and the only gene associated with barrier function whose expression was affected by the HG feeding was *ZO-1*. This gene was consistently identified as an important discriminant for the HG challenge by the sPLS-DA analysis, regardless of the microbial niche included in the model. Therefore, the expression of *ZO-1* in the rumen epithelium seems to be a strong indicator of the health status of the organ. After reaching the lowest relative expression on week 2 HG, *ZO-1* expression increased on the third week of HG feeding suggesting that the barrier integrity can be restored even if impaired in the first weeks of a HG challenge. The negative correlation between *IGF-1* and *ZO-1* might imply a potential disruption of the epithelial barrier due to the excessive papillae growth. However, by the end of the experiment, the metabolic capacity of the microbiota has adapted to the levels of starch in the diet and can metabolize it faster, limiting the growth and helping to maintain the integrity of the barrier. In fact, our findings for the digesta microbiota seem to suggest that the rumen started adapting by the fourth week of continuous HG feeding. In particular, in LAM, that represents the microbial community loosely associated with feed particles (Tafaj et al. 2004), the beta diversity showed a significant number of samples from the last week of experiment clustering together with the samples collected in forage feeding. This means that, in some individuals, the microbiota of this niche by the last week of experiment had switched to a composition more similar to the first week of experiment. In an analogous study that analyzed the effects of rhubarb powder on the rumen in cows fed a HG diet, the microbiota composition recovered by the end of the experiment, suggesting an adaptation of the microorganisms to changing conditions (Wang et al. 2017). Furthermore, studies in beef cattle have demonstrated that the ruminal microbiota starts to shift toward a stable composition by the fourth week after a change in dietary regime, and that such modifications are stable over time (Clemmons et al. 2019, Snelling et al. 2019). It is interesting to notice that, as it is known from shorter studies, also in our prolonged dietary challenge the three ruminal microenvironments showed very different adaptations, and the LAM microbiota seemed to be the first to start the adaptation process (McCann et al. 2016, Ricci et al. 2022). The results of the multiblock sPLS-DA analyses also revealed a strong correlation between this ruminal niche and the rumen metabolome, highlighting the crucial role of LAM in the ruminal metabolic adaptation.

At the metabolome level, the changes in concentration fluctuated over the weeks. Hierarchical clustering evidenced a similarity between the metabolite concentration on week 1 HG and week 3 HG, and between week 2 HG and week 4 HG. The variation in the metabolome in the first week of HG coincided with substantial changes in the microbiota composition, which indicate the instauration of ruminal dysbiosis, as discussed above. Even though some metabolites and biogenic amines showed lower concentrations during the second week of HG, after this time the system seemed to collapse, aggravating the dysbiosis. This breaking point coincided with a considerable shift in microbiota composition, as shown in the beta diversity graphs, and with the lowest alpha diversity values, as well as with the highest concentration of harmful compounds. Finally, in the last week of experiment, both the microbial composition and activity seemed to stabilize, with numerically higher diversity and richness indices and lower metabolite concentrations. All the evidence gathered suggests that the ruminal microbiota can restore its composition and activity during the course of a long term HG challenge, by optimizing the utilization of substrates introduced with continuous HG feeding, and at the same time preserving the integrity of the epithelium (Weimer 2015).

### Effects of the phytogenic supplementation on the ruminal microbiota composition and the epithelial inflammatory response

The PFA supplementation contributed to maintain a stable microbiota composition of the epimural fraction during the HG challenge, while it affected specific taxa in the rumen digesta. [*Eubacterium*] *xylanophilum* group, *Lachnospiraceae* NC2004 group and uncultured *Peptococcaceae* were affected in a similar way both in LAM and SAM, but there is lack of research on the effects of phytogenic compounds on these taxa. *Coprococcus* 2 and *Lachnospiraceae* NC2004 group, which were both reduced in the PFA group, have been related to the metabolization of polyphenols (Patel et al. 1981, Burgos-Edwards et al. 2018, Liu et al. 2020). [*Eubacterium*] *xylanophilum* group can metabolize only a few carbohydrates, and preferentially contributes to the degradation of hemicellulose (Van Gylswyk and Van Der Toorn 1985). The increased relative frequency of *Peptococcaceae* in the rumen contents might indicate the capacity of the PFA supplementation to preserve a physiological composition of the microbiota, since it has been demonstrated that this family is negatively affected by the presence of starch (Kheirandish et al. 2022). Furthermore, the lower number of taxa affected by the prolonged HG feeding in the PFA group compared to the control, confirms the potential of the phytogenic supplementation to preserve a more stable microbiota composition in the rumen during a prolonged dietary challenge.

Nevertheless, the effects of the PFA supplementation on the relative expression of some genes in our study might be the result of shifts in microbiota composition due to the treatment. Interestingly, the PFA supplementation decreased the teichoic acid biosynthesis predicted pathway (TEICHOICACID-PWY) as well as the relative frequency of Gram-positive bacteria such as *Coprococcus* 2, *Ruminococcus* 1, and [*Eubacterium*] *xylanophilum* group. This was probably related to the lower expression of *TLR-2* in the PFA group. In fact, *TLR-2* reacts to Gram-positive bacteria, but its role in the interaction with the proliferation of specific taxa especially in HG feeding regimes requires further research (Takeuchi et al. 1999, Petri et al. 2019). Our integrative analysis did not show any correlations between this gene and specific ASVs, although it revealed a positive correlation with α- and β-aminobutyric acid, which were also identified as main drivers of difference between the control and the PFA group in EAM. While α-aminobutyric acid is known to be an agonist for *TLR-2* (Santone et al. 2015), the role of the β isomer in relation to inflammation in cattle is not well known. β-aminobutyric acid is produced in plants in response to stressing stimuli, therefore the higher concentration of this metabolite in the PFA group might be due to its presence in the phytogenic supplement itself (Thevenet et al. 2017). However, β-aminobutyric acid was also positively correlated with several ASVs with very different taxonomic classification, and its concentration was reduced in weeks 1 and 2 HG, suggesting its faster metabolization by the ruminal bacteria for the production of proteins (Maeng et al. 1976, Thevenet et al. 2017).

In goats fed highly fermentable diet, the proinflammatory cytokine *IL-6* decreased in parallel with the anti-inflammatory cytokine *IL-10* (Shen et al. 2016). Similarly, in our study both cytokines showed the same trend due to PFA supplementation, although they were not affected by the dietary regime. Furthermore, the PFA treatment also decreased the expression of *MyD88*, although not significantly. It is suggested that this expression pattern might be aimed at incrementing the host tolerance toward some bacterial strains that proliferate when the animals are fed HG diets (Shen et al. 2016). *MyD88* was in fact identified as one of the main discriminants between control and PFA group in all the sPLS-DA models and showed correlations with taxa significantly increased by the HG diet, such as *Prevotellaceae, Lachnospiraceae*, and *Ruminococcaceae*. Furthermore, it needs to be considered that the production of *IL-6* might also activate anti-inflammatory pathways: it has been suggested that the expression of this cytokine is aimed at restoring the homeostasis by controlling the inflammatory response (Xing et al. 1998). Interestingly, the acute phase proteins measured in our study were reduced by the PFA supplementation, as reported in our companion study (Rivera-Chacon et al. 2022). Although the anti-inflammatory effects of secondary plant compounds have been reported before in cattle and other species, the mechanisms of action of these blends have not been elucidated yet (Petri et al. 2020, Latek et al. 2022, Wang et al. 2023).

### Effects of the phytogenic supplementation on the microbial metabolism and ruminal epithelium structure

Although the PFA treatment affected some relatively high abundant taxa, it did not significantly impact the overall ruminal microbiota composition, as shown by the microbial diversity. On the other hand, the phytogenic supplementation showed effects on the microbial predicted activity and on the measured metabolites. This might be explained as the phytogenics are more effective toward the microbial metabolism, rather than affecting microbiota composition (Hassan et al. 2020).

In fact, the PFA treatment impacted several metabolites and biogenic amines in our study. For example, the stabilization of concentration of 5-AVA over the weeks could prevent the formation of 5-aminovaleric acid betaine, a potentially harmful compound derived from the fermentation of 5-AVA by the microbiota (Haikonen et al. 2022). The increment in methylbutyric acid due to the PFA, especially in the last weeks of experiment, could improve the fermentation in the rumen, as supported by previous studies in which the supplementation with 2-methylbutyric acid promoted the growth of cellulolytic bacteria *in vitro* and improved feed digestion in beef cattle (Dehority et al. 1967, Wang et al. 2012). Additionally, the PFA treatment maintained stable lower levels of dopamine, which might contribute to increase rumen contractions, since such metabolite has been demonstrated to reduce rumino-reticular motility in sheep (Buéno et al. 1983). This, in turn, could enhance the clearance of VFAs and contribute to the buffering of ruminal pH (Rivera-Chacon et al. 2022).

Similarly, the PFA supplementation reduced the concentration of kynurenine, a potentially harmful metabolite derived from the metabolization of tryptophan (Mándi and Vécsei 2012, Bae et al. 2020). The production of kynurenine is stimulated by several cytokines, including *IFN-γ* (Mándi and Vécsei 2012), but the lower concentrations measured in the rumen did not correspond to a reduced epithelial expression of interferon in the PFA group. The PFA supplementation also increased the L-tryptophan biosynthesis pathway (TRPSYN-PWY) in LAM samples, but it is possible that the metabolism of kynurenine at the epithelial level was not shifted toward the production of toxic metabolites. It has in fact been observed that most of the ingested tryptophan is processed in the intestinal epithelium as well as in the liver through the kynurenine pathway, which results in *de novo* NAD^+^ biosynthesis (Castro-Portuguez and Sutphin 2020). Dysregulation of tryptophan–kynurenine metabolism and NAD^+^ synthesis may promote mitochondrial malfunction, and consequently alter the production of ATP (Castro-Portuguez and Sutphin 2020, Castro-Portuguez et al. 2020). It is known that the tightness of the epithelial barrier of the gastrointestinal tract is strictly ensured by a correct expression of *ZO-1*, whose activity is ensured by a constant production of ATP by the mitochondria (Rossi 2022). In our study, the immunohistochemical evaluations showed that the intrinsic apoptotic pathway was not activated in the ruminal epithelial cells, suggesting that the mitochondrial activity was preserved. Thus, the physiological expression of tight junctions was maintained, ensuring the integrity of the epithelial barrier of the rumen (Steele et al. 2011, McCann et al. 2016). Considering this, although our model did not show a significant impact of the PFA supplementation on the relative expression of *ZO-1*, the more stable concentration of kynurenine observed in the PFA group might have helped to preserve a regular expression of this tight junction.

Studies in other species demonstrated the potential beneficial effects of phytogenic compounds on the gut barrier function (Bachinger et al. 2019, Latek et al. 2022) and the PFA supplementation in our study reduced the relative expression of *CLDN-4* and *DSG1*. However, this is in contrast with previous findings in cattle, in which phytogenic substances did not impact genes related with the barrier function (Petri et al. 2020), indicating that other elements, such as the experimental design, might have contributed to the altered gene expression in the rumen. Furthermore, the relevance network showed a correlation between *CLDN4* and glyceric acid. This metabolite was reduced by the PFA supplementation and is part of the pentose phosphate pathway, implying a possible correlation between *CLDN4* expression and the utilization of starch in the rumen.

The integrative analysis performed with the treatment as explanatory variable provided results largely overlapping with the findings for the model considering the weeks. This might indicate that the strong influence of the prolonged HG feeding on the sPLS-DA model could possibly mask some of the effects of the PFA. Further research is warranted to establish the mechanisms of action of the phytogenic supplementation on the ruminal microbiota and epithelial structure.

### Conclusion

The prolonged feeding of HG diet caused ruminal dysbiosis, which was noticeable in the rumen digesta, both in LAM and SAM, especially after the second week of HG feeding, and decreased in severity by the last week of experiment. The increased expression of genes related with inflammation and with epithelial growth indicated a reaction of the epithelium to the challenging conditions. The period between weeks 2 and 3 HG was recognized as the breaking point for the homeostasis of the ruminal ecosystem, and our integrative analysis identified *ZO-1, MyD88* and *Prevotella* 1 as main drivers for the ruminal response. The changes in barrier integrity genes as well as the lack of significant alterations of the rumen wall structure by the fourth week of experiment suggest a process of adaptation of the ruminal environment to the prolonged HG feeding. The metabolic adaptation of the rumen to the diet seems to be led mostly by the microbial activity of the LAM fraction. The PFA supplementation showed the potential to aid this adaptive process by altering the microbial activity, specifically reducing harmful metabolites, such as dopamine and 5-AVA, and by shifting the epithelial gene expression to increase the tolerance toward the microbiota, through the alteration of the expression of *TLR-2, IL-6*, and *IL-10*. Future studies should be aimed at investigating the mechanism of action of the phytogenic compounds.

## Supplementary Material

fiae006_Supplemental_Files

## Data Availability

Sequences and relative information are available at the NCBI database BioProject PRJNA802085.
