# Supplementary material for: Integrated microbiota–host–metabolome approaches reveal adaptive ruminal changes to prolonged high-grain feeding and phytogenic supplementation in cattle
Source: FEMS Microbiol Ecol. 2024 Jan 27;100(2):fiae006. doi: 10.1093/femsec/fiae006 (PMC10858391; doi:10.1093/femsec/fiae006)

**Supplementary Table 1.** Good’s coverage index calculated for solid associated microbiota (SAM), liquid associated microbiota (LAM), and epithelial adherent microbiota (EAM), after rarefaction. Mean values and standard error of the mean are presented at the baseline and per each week of high grain feeding (HG) for control and treatment (PFA) group.

|  | Forage - baseline | | 1 HG | | | 2 HG | | | 3 HG | | | 4 HG | | | P-values^1^ | | | |  |
| --- | --- | --- | --- | --- | --- | --- | --- | --- | --- | --- | --- | --- | --- | --- | --- | --- | --- | --- | --- |
|  | **Control** | **PFA** | | **Control** | **PFA** | | **Control** | **Treatment** | | **Control** | **PFA** | | **Control** | **PFA** | | **PFA** | **Diet** | **I** | |
| SAM | 0.93 ± 0.01 | 0.94 ± 0.01 | | 0.96 ± 0.01 | 0.96 ± <0.01 | | 0.97 ± <0.01 | 0.96 ± <0.01 | | 0.96 ± 0.01 | 0.97 ± <0.01 | | 0.96 ± <0.01 | 0.96 ± 0.01 | | 0.96 | < 0.01 | 0.57 | |
| LAM | 0.93 ± <0.01 | 0.93 ± 0.01 | | 0.97 ± <0.01 | 0.95 ± 0.01 | | 0.96 ± 0.01 | 0.97 ± 0.01 | | 0.97 ± <0.01 | 0.97 ± 0.01 | | 0.95 ± 0.01 | 0.95 ± 0.01 | | 0.46 | < 0.01 | 0.98 | |
| EAM | 0.95 ± 0.01 | 0.96 ± 0.01 | | 0.96 ± 0.01 | 0.96 ± <0.01 | | 0.96 ± <0.01 | 0.96 ± <0.01 | | 0.95 ± <0.01 | 0.95 ± <0.01 | | 0.95 ± <0.01 | 0.95 ± 0.01 | | 0.75 | 0.21 | 0.84 | |

^1^P-values for the effect of phytogenic treatment (PFA), diet within week (Diet) and of the interaction between treatment within week and diet (I).

**Supplementary Table 2.** Indices measured with histological and immunohistochemical evaluations. (TUNEL = Apoptosis index; PCK = Pan-cytokeratin; SC = Thickness of the stratum corneum).

|  |  |  | | P-values^1^ | | | | |
| --- | --- | --- | --- | --- | --- | --- | --- | --- |
|  | **Forage** | | **HG** | | **Cow** | **PFA** | **Diet** | **I** |
| TUNEL | 23.1 ± 1.6 | | 26.5 ± 1.5 | | 0.68 | 0.26 | 0.64 | 0.34 |
| PCK | 100 ± 4 | | 108 ± 3 | | 0.53 | 0.53 | 0.23 | 0.87 |
| SC | 27.1 ± 2.5 | | 33.5 ± 2.7 | | 0.10 | 0.72 | 0.24 | 0.14 |

^1^P-values for the effect of the single animal (Cow), phytogenic treatment (PFA), diet within week (Diet) and of the interaction between treatment within week and diet (I).

**Supplementary Table 3.** Correlation coefficients obtained with the sPLS-DA analysis per each pair of datasets and per each component with experimental week as explanatory variable. The models were run for the three microbial datasets (SAM = solid associated microbiota, LAM = liquid associated microbiota, and EAM = epithelial adherent microbiota) in association with metabolome and gene expression (GE).

|  | Week | | | | | | | | |
| --- | --- | --- | --- | --- | --- | --- | --- | --- | --- |
| LAM | Component 1 | Component 2 | Component 3 | Component 4 | Component 5 | Component 6 | Component 7 | Component 8 | Component 9 |
| Metabolome-Microbiota | 0.82 | 0.72 | 0.51 | 0.68 | 0.67 | 0.59 | 0.51 | 0.72 | 0.65 |
| GE-Microbiota | 0.23 | 0.53 | 0.68 | 0.3 | 0.45 | 0.38 | 0.45 | 0.34 | 0.27 |
| GE-Metabolome | 0.38 | 0.42 | 0.2 | 0.18 | 0.25 | 0.16 | 0.23 | 0.32 | 0.14 |
| SAM | Component 1 | Component 2 | Component 3 | Component 4 | Component 5 | Component 6 | Component 7 | Component 8 | Component 9 |
| Metabolome-Microbiota | 0.71 | 0.76 | 0.4 | 0.82 | 0.59 | 0.62 | 0.63 | 0.56 | 0.63 |
| GE-Microbiota | 0.55 | 0.51 | 0.76 | 0.43 | 0.33 | 0.41 | 0.62 | 0.39 | 0.29 |
| GE-Metabolome | 0.39 | 0.34 | 0.23 | 0.42 | 0.23 | 0.26 | 0.15 | 0.2 | 0.23 |
| EAM | Component 1 | Component 2 | Component 3 | Component 4 | Component 5 | Component 6 | Component 7 | Component 8 | Component 9 |
| Metabolome-Microbiota | 0.73 | 0.66 | 0.74 | 0.54 | 0.55 | 0.68 | 0.48 | 0.64 | 0.58 |
| GE-Microbiota | 0.6 | 0.58 | 0.33 | 0.33 | 0.31 | 0.25 | 0.66 | 0.38 | 0.55 |
| GE-Metabolome | 0.43 | 0.26 | 0.35 | 0.16 | 0.37 | 0.12 | 0.36 | 0.22 | 0.15 |

**Supplementary Table 4.** Correlation coefficients obtained with the sPLS-DA analysis per each pair of datasets and per each component with experimental treatment (PFA) as explanatory variable. The models were run for the three microbial datasets (SAM = solid associated microbiota, LAM = liquid associated microbiota, and EAM = epithelial adherent microbiota) in association with metabolome and gene expression (GE).

|  | PFA | | | | | | | | |
| --- | --- | --- | --- | --- | --- | --- | --- | --- | --- |
| LAM | Component 1 | Component 2 | Component 3 | Component 4 | Component 5 | Component 6 | Component 7 | Component 8 |  |
| Metabolome-Microbiota | 0.82 | 0.69 | 0.65 | 0.61 | 0.58 | 0.63 | 0.66 | 0.62 |  |
| GE-Microbiota | 0.23 | 0.61 | 0.38 | 0.39 | 0.45 | 0.37 | 0.33 | 0.35 |  |
| GE-Metabolome | 0.39 | 0.36 | 0.3 | 0.23 | 0.21 | 0.34 | 0.32 | 0.34 |  |
| SAM | Component 1 | Component 2 | Component 3 | Component 4 | Component 5 | Component 6 | Component 7 | Component 8 |  |
| Metabolome-Microbiota | 0.75 | 0.67 | 0.59 | 0.51 | 0.47 |  |  |  |  |
| GE-Microbiota | 0.71 | 0.63 | 0.61 | 0.43 | 0.86 |  |  |  |  |
| GE-Metabolome | 0.46 | 0.41 | 0.39 | 0.12 | 0.35 |  |  |  |  |
| EAM | Component 1 | Component 2 | Component 3 | Component 4 | Component 5 | Component 6 | Component 7 | Component 8 |  |
| Metabolome-Microbiota | 0.76 | 0.58 | 0.39 | 0.65 | 0.7 | 0.61 | 0.4 | 0.67 |  |
| GE-Microbiota | 0.62 | 0.63 | 0.81 | 0.4 | 0.35 | 0.39 | 0.78 | 0.16 |  |
| GE-Metabolome | 0.44 | 0.22 | 0.4 | 0.27 | 0.24 | 0.28 | 0.21 | 0.31 |  |

**Supplementary Figure 1.** Bar-graph showing the mean relative frequency of the most abundant phyla across solid associated microbiota (SAM) (**A**), liquid associated microbiota (LAM) (**B**), and epithelial adherent microbiota (EAM) (**C**) samples. Results are presented for the control and the treatment (PFA) groups per each experimental week.


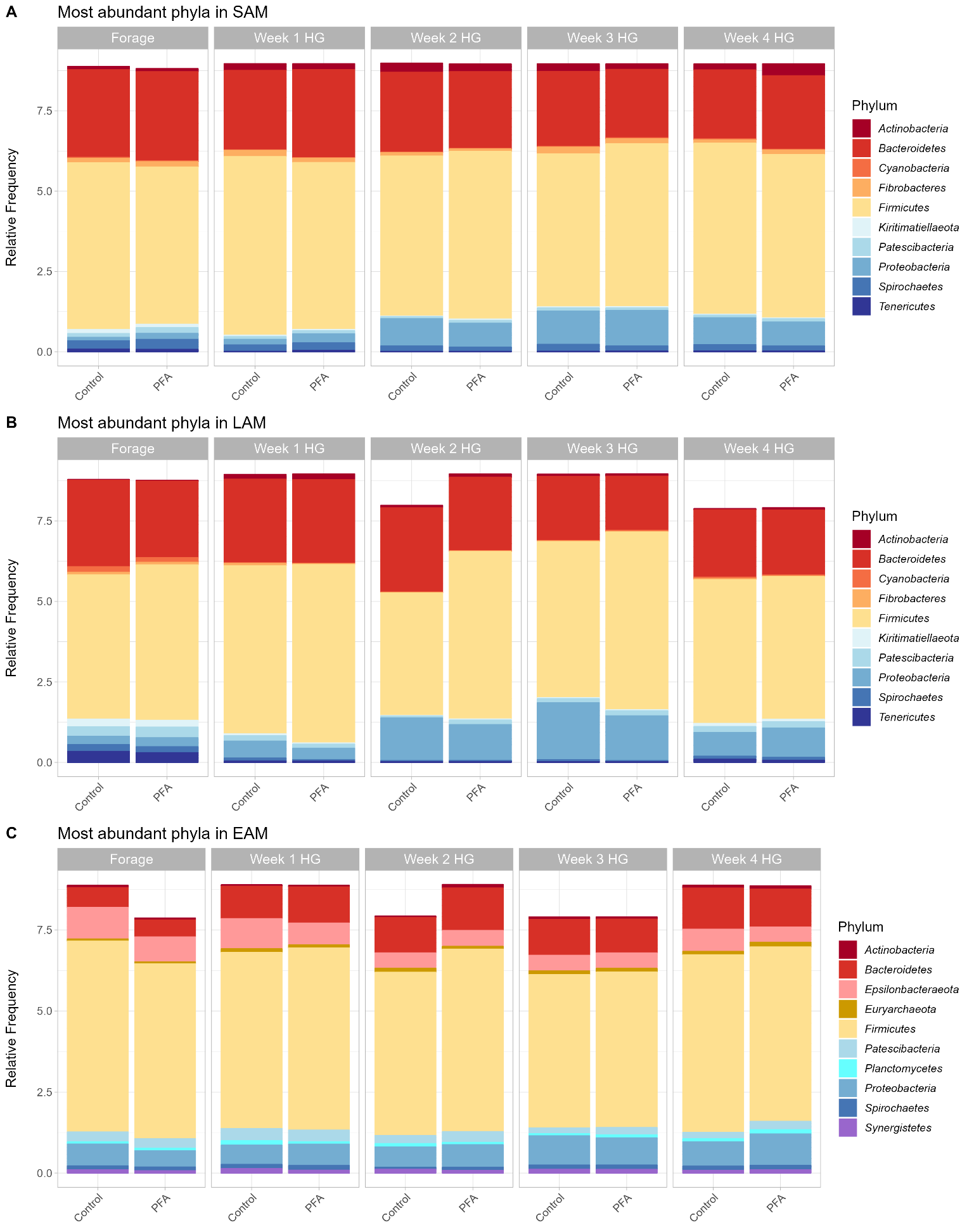


**Supplementary Figure 2.** Effect of the PFA supplementation on the PICRUSt2 predicted pathways in solid associated microbiota (SAM) and liquid associated microbiota (LAM) samples. The effect size reported (log2 fold change) is based on the output of the differential abundance analyses ran with MaAsLin2 package in R.


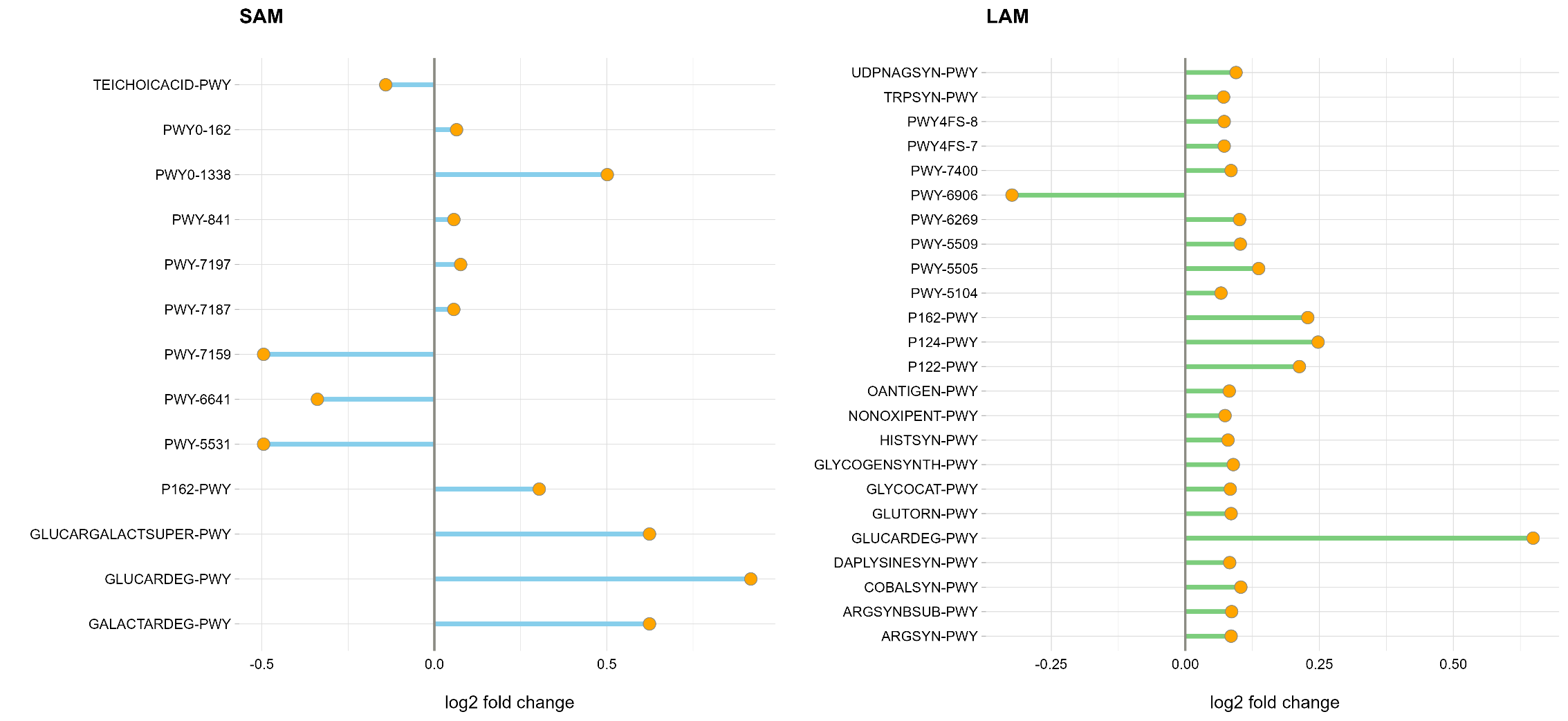


**Supplementary Figure 3.** Principal Components Analysis (PCA) of the variance of the metabolite composition in the rumen fluid based on normalized values. PCA loadings detected confirmed the three major VFAs (acetic acid, propionic acid and butyric acid) as major responsible for the metabolites distribution variation between the weeks.


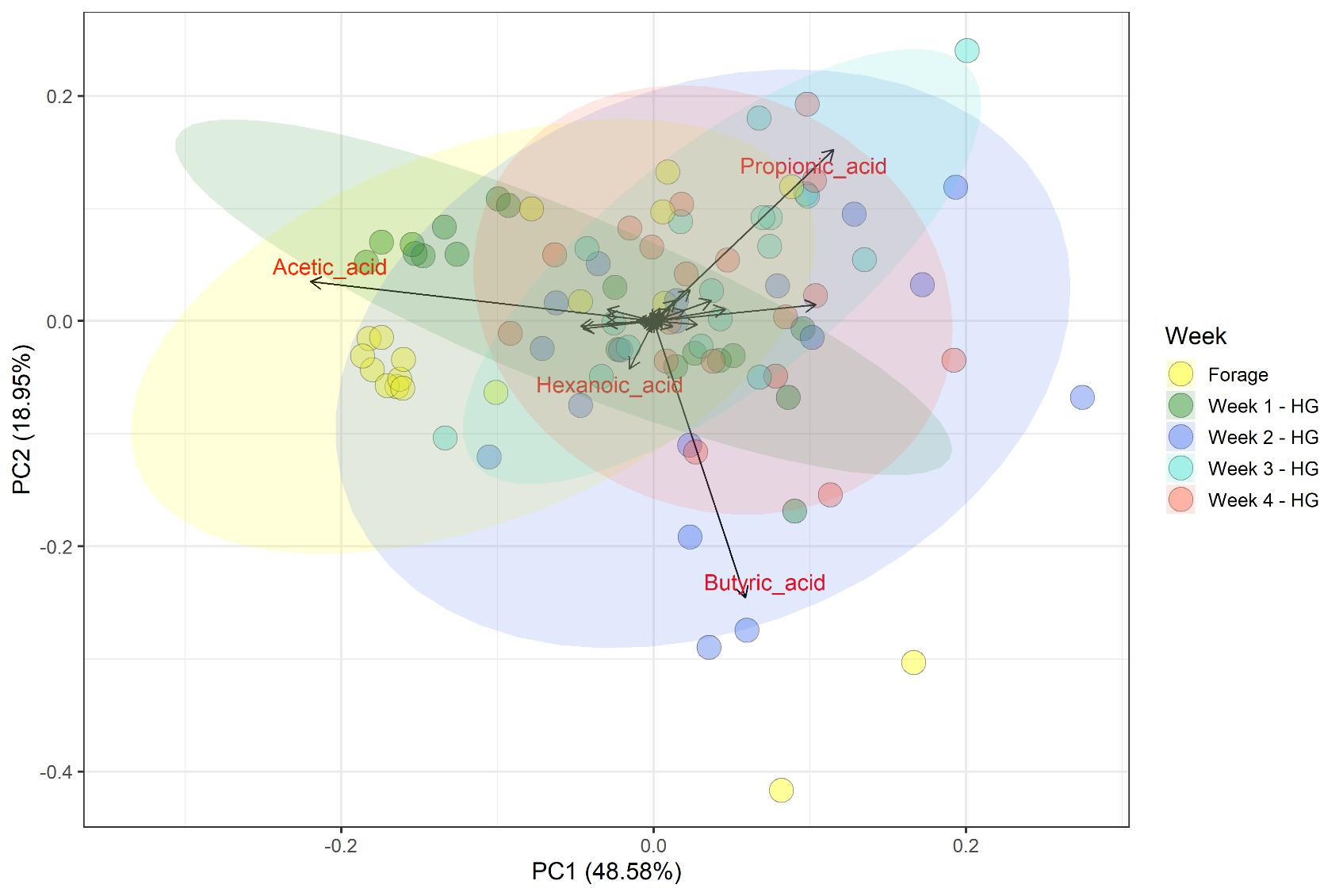


**Supplementary Figure 4.** Histological sections of ruminal papillae of cows fed a forage diet (**A**) or a high-grain diet for four consecutive weeks (**B**) stained with DeadEnd™ Colorimetric TUNEL System (Promega Italia Srl, Italy) for the evaluation of cellular apoptosis.


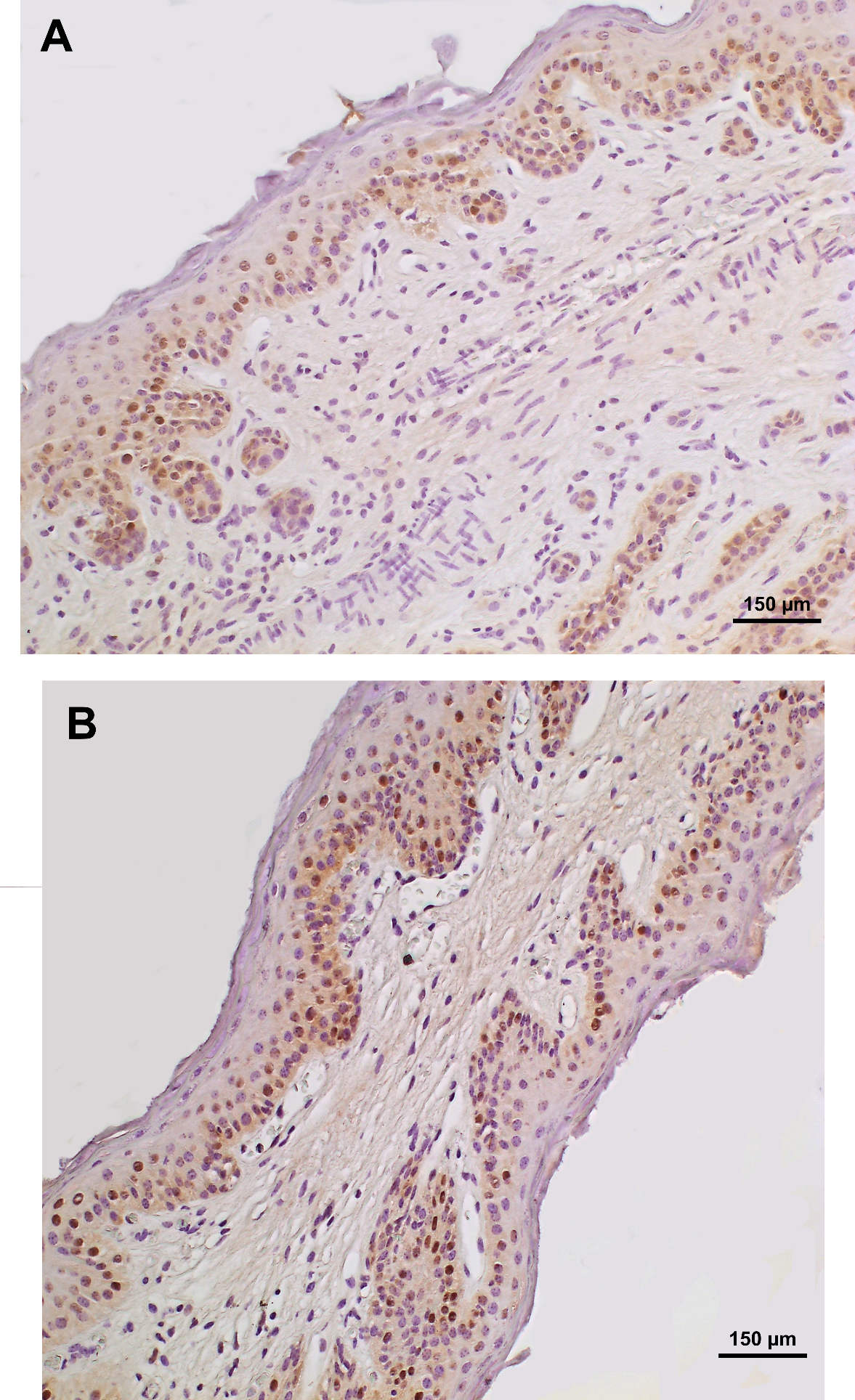


**Supplementary Figure 5.** Histological sections of ruminal papillae of cows fed a forage diet (**A**) or a high-grain diet for four consecutive weeks (**B**) immunostained with monoclonal mouse anti-human Cytokeratin antibody, clone AE1/AE3 (diluted 1:200, Agilent, USA), to evaluate the thickness of the keratin layer and to measure the thickness of the stratum corneum.


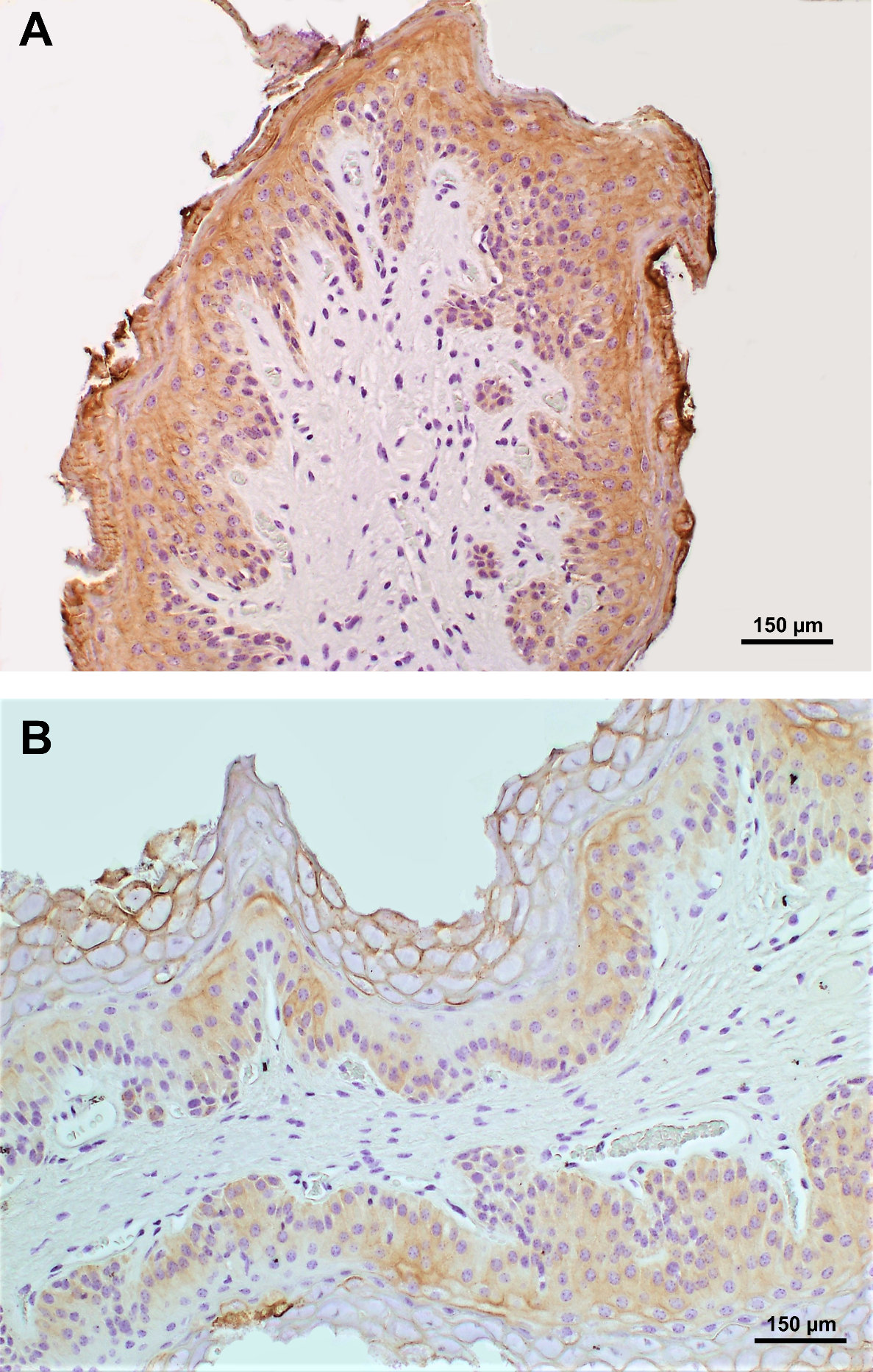


**Supplementary Figure 6.** Loading plots for solid associated microbiota (SAM) showing the maximum contributions on each component for gene expression, metabolome, and microbiota (ASVs), respectively. The results are presented for the models run with experimental week (Week) and with the treatment (PFA) as explanatory variables. Each color represents a different component.


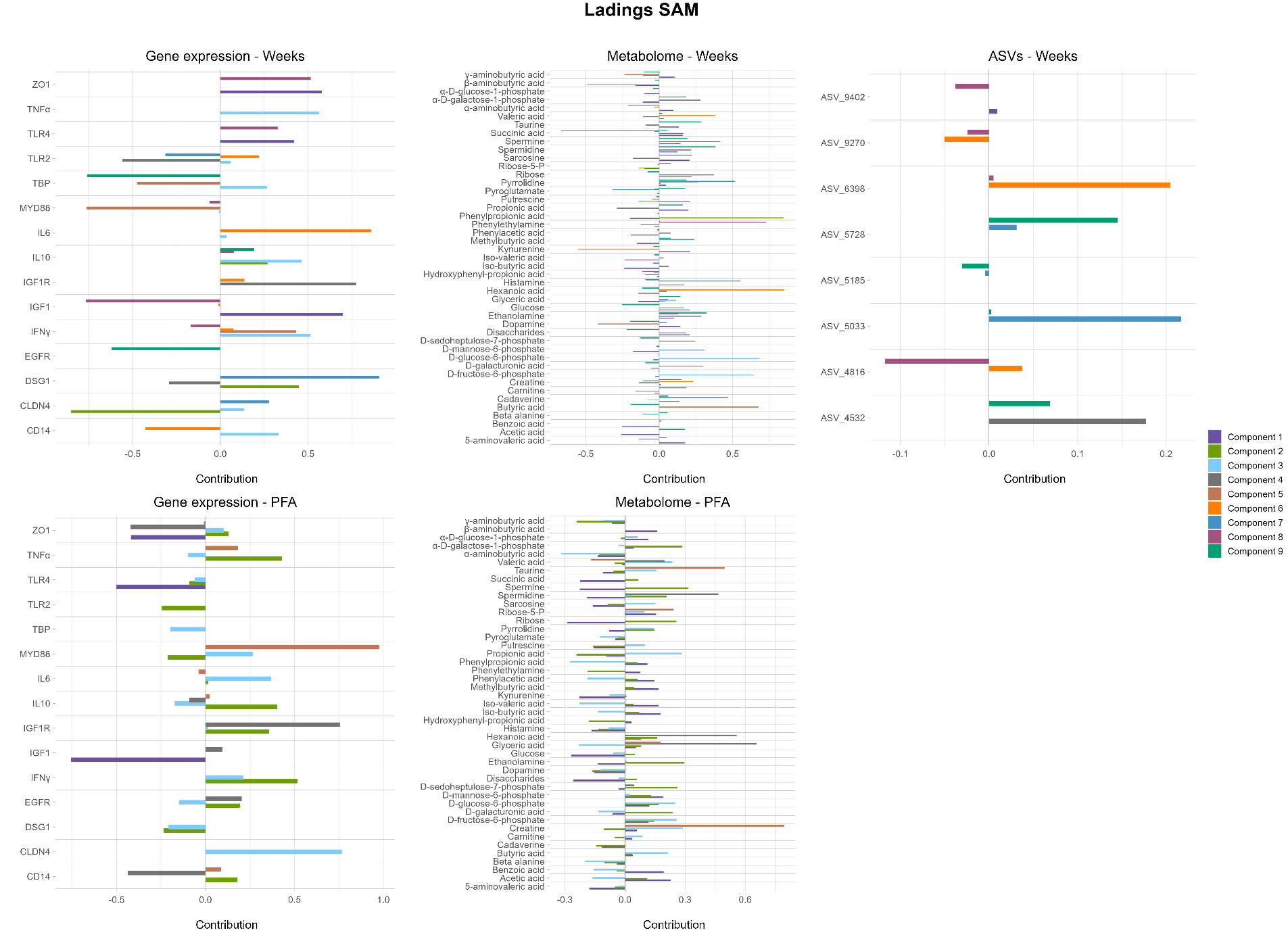


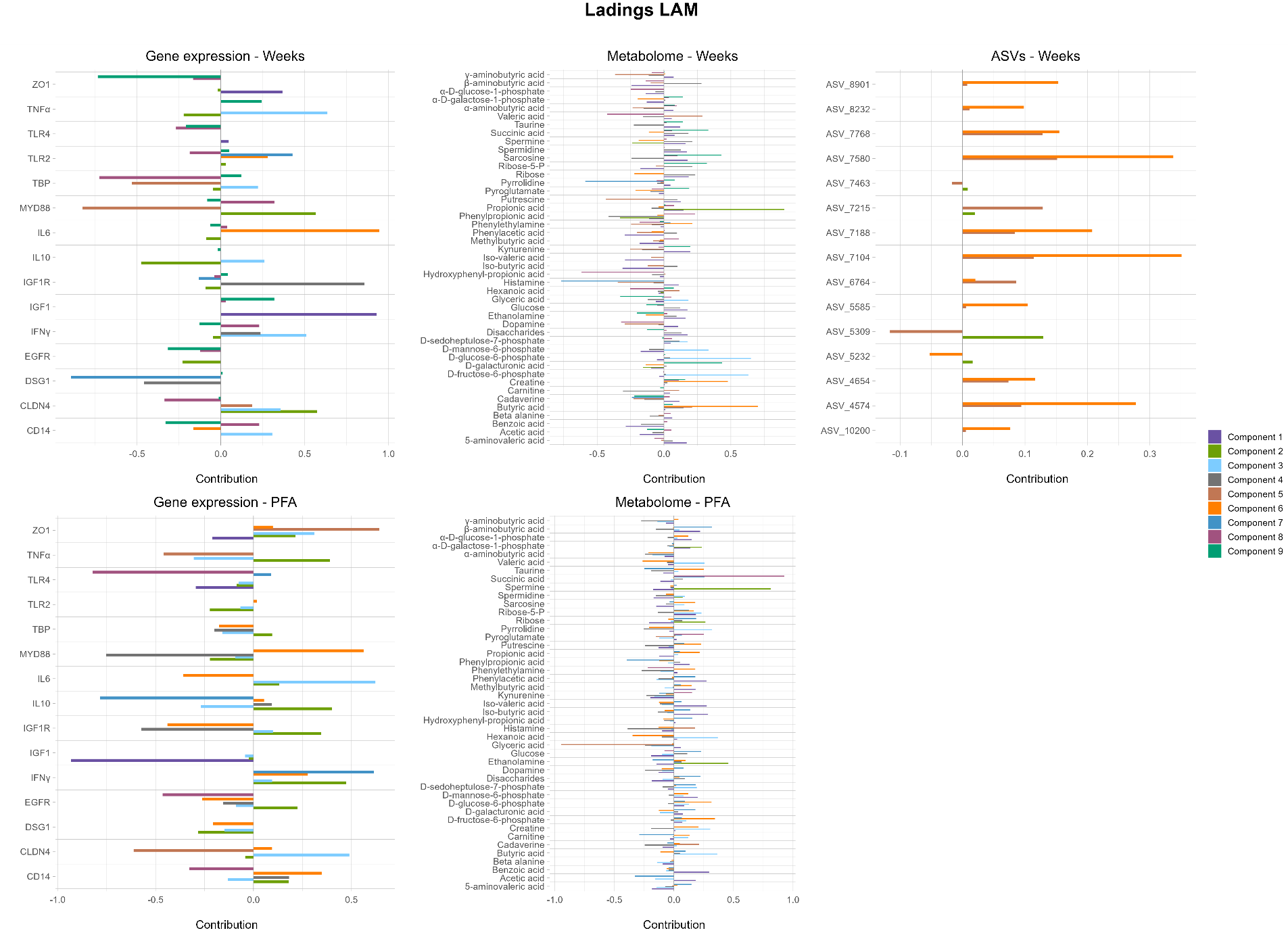
**Supplementary Figure 7.** Loading plots for liquid associated microbiota (LAM) showing the maximum contributions on each component for gene expression, metabolome, and microbiota (ASVs), respectively. The results are presented for the models run with experimental week (Week) and with the treatment (PFA) as explanatory variables. Each color represents a different component.

**Supplementary Figure 8.** Loading plots for epithelial adherent microbiota (EAM) showing the maximum contributions on each component for gene expression, metabolome, and microbiota (ASVs), respectively. The results are presented for the models run with experimental week (Week) and with the treatment (PFA) as explanatory variables. Each color represents a different component.


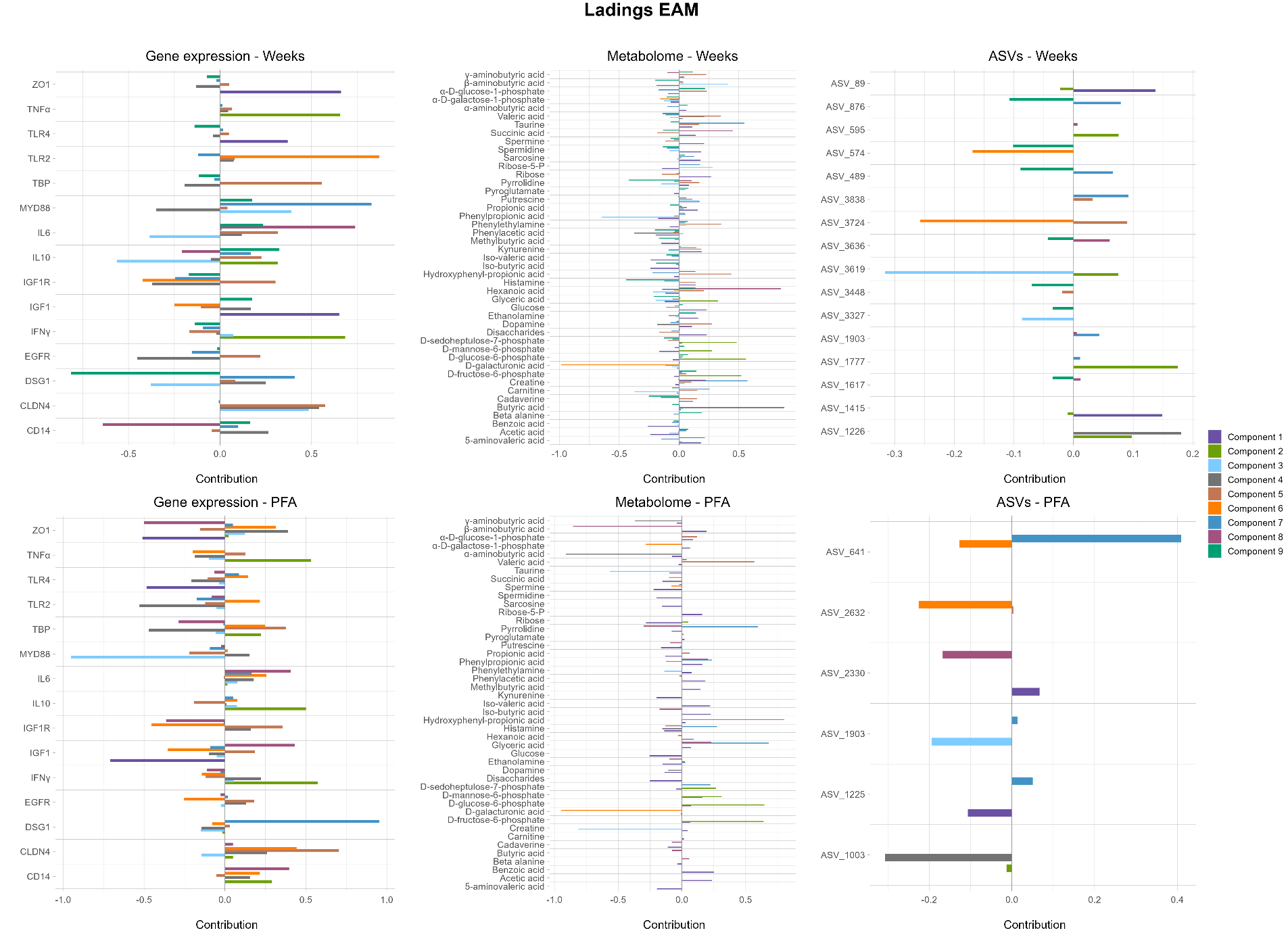

Supplement: fiae006_Supplemental_Files [file fiae006_supplemental_files.zip › FEMS paper Ricci - Supplementary material_15.01.24.docx]
